# Effect of ammonia and water molecule on OH + CH_3_OH reaction under tropospheric condition

**DOI:** 10.1038/s41598-021-90640-6

**Published:** 2021-06-09

**Authors:** Mohamad Akbar Ali, M. Balaganesh, Faisal A. Al-Odail, K. C. Lin

**Affiliations:** 1 Department of Chemistry, College of Science, King Faisal University, PO Box 380, Al Hufuf, 31982 Al-Ahsa Saudi Arabia; 2 Department of Chemistry, National Taiwan University, Taipei, Taiwan

**Keywords:** Atmospheric chemistry, Computational chemistry, Homogeneous catalysis, Chemistry, Environmental chemistry

## Abstract

The rate coefficients for OH + CH_3_OH and OH + CH_3_OH (+ X) (X = NH_3_, H_2_O) reactions were calculated using microcanonical, and canonical variational transition state theory (CVT) between 200 and 400 K based on potential energy surface constructed using CCSD(T)//M06-2X/6-311++G(3df,3pd). The results show that OH + CH_3_OH is dominated by the hydrogen atoms abstraction from CH_3_ position in both free and ammonia/water catalyzed ones. This result is in consistent with previous experimental and theoretical studies. The calculated rate coefficient for the OH + CH_3_OH (8.8 × 10^−13^ cm^3^ molecule^−1^ s^−1^), for OH + CH_3_OH (+ NH_3_) [1.9 × 10^−21^ cm^3^ molecule^−1^ s^−1^] and for OH + CH_3_OH (+ H_2_O) [8.1 × 10^−16^ cm^3^ molecule^−1^ s^−1^] at 300 K. The rate coefficient is at least 8 order magnitude [for OH + CH_3_OH(+ NH_3_) reaction] and 3 orders magnitude [OH + CH_3_OH (+ H_2_O)] are smaller than free OH + CH_3_OH reaction. Our calculations predict that the catalytic effect of single ammonia and water molecule on OH + CH_3_OH reaction has no effect under tropospheric conditions because the dominated ammonia and water-assisted reaction depends on ammonia and water concentration, respectively. As a result, the total effective reaction rate coefficients are smaller. The current study provides a comprehensive example of how basic and neutral catalysts effect the most important atmospheric prototype alcohol reactions.

## Introduction

Methanol (CH_3_OH) is most abundant volatile organic compounds (VOCs) in the atmosphere. The main sources of the emission of CH_3_OH are from living organisms and human activities^1–3^. CH_3_OH has been used as a fuel additive to reduce emissions of hydrocarbons from automobile^1–3^. The reactions of CH_3_OH may have elusive and indirect effects in the formation of secondary organic aerosols, therefore their impact on the tropospheric oxidizing power, disturbing air quality and adverse effect on human health^1–3^. CH_3_OH abundances are also dominated by direct emissions, but some sources also include the oxidation pathways of methane and from other volatile organic species. The oxidation of methanol forms species that includes carbon monoxide (CO), formaldehyde (HCHO), and tropospheric ozone (O_3_). Because the reaction of hydroxyl radical (OH) with CH_3_OH is the most important sink for this simplest alcohol; it is therefore determining the atmospheric lifetime of CH_3_OH.

Many experimental and theoretical studies on the OH + CH_3_OH reaction system have been performed^1–5^. As suggested in earlier works^1–5^, OH + CH_3_OH proceeds hydrogen abstraction by the OH radical at either the methyl (–CH_3_) or the hydroxyl site (–OH) of CH_3_OH, assisted by the formation of a pre-reactive complex^3,4^. It is known that hydrogen abstraction from CH_3_ group is the most dominated channel than the hydrogen abstraction from OH site at temperature ≥ 200 K^3,4^. Recently, Nguyen et al.^5^ reported reaction rate coefficients for OH + CH_3_OH reaction at temperature ≤ 200 K. They suggested that the formation of CH_3_O radical plays a more important role due to quantum mechanical tunneling.

Gaseous ammonia (NH_3_) is the most abundant alkaline gas in the atmosphere. The main source of NH_3_ emissions is agriculture, including animal husbandry and NH_3_-based fertilizer applications^6–8^. It has been suggested that NH_3_ emissions have been increasing over the last few decades on a global scale. Besides, its concentration in atmosphere, ammonia emission is steadily increasing from the food production industries^6–8^. It is expected that global emission of ammonia could increase by many folds in future. It is known that ammonia molecules present in the atmosphere can influence the many important atmospheric reactions^6–10^. For this purpose, it is important to have a clear understanding of OH radical reaction with CH_3_OH in presence of NH_3_. NH_3_ has similar efficiency as H_2_O in catalyzing many hydrogen abstraction reactions. Based on that, the possibility of the NH_3_-catalytic effect on the HO + CH_3_OH reaction has been investigated in this work.

To the best of our knowledge, there is only one theoretical study reported by Jonas et al. on the catalytic effect of NH_3_ on the OH + CH_3_OH reaction^9^. They have investigated the mechanism and kinetics of the OH + CH_3_OH reaction with and without the presence of H_2_O, NH_3_, and H_2_SO_4_ using ab initio/DFT. They concluded that participation of NH_3_ on the OH + CH_3_OH reaction could enhance the reaction rate and change the reaction mechanism, but this process is unlikely to occur under the atmospheric conditions, due to a very weak interaction NH_3_ with CH_3_OH.

During the past few years, many research groups have proposed the catalytic effect of a single water molecule for many atmospheric reactions^11–26^. The possibility of one water molecule as a catalyst in reactions of OH + CH_3_OH, have been proposed by Jara et al.^12,13^ and Chao et al. Chao et al.^11^ have measured reaction rate coefficients with water molecule and suggested that no catalytic role of water molecule was observed, which contradict the measurement of Jara-Toro et al.^12,13^ Recently, Wu et al.^14^ have proposed the detailed study of water-assisted pathways and predicted temperature-dependent rate coefficients for OH + CH_3_OH (+ H_2_O) using hybrid functional coupled with advanced kinetic models^14^. The result reported by them is in ∼1–2 order magnitude smaller than reaction without water. In our previous works, we revealed that the catalytic effect of H_2_O molecules is not important in the atmospheric reactions *i.e.,* OH + CH_2_O and OH + CH_2_CH_2_, OH + CH_2_NH and CH_2_NH + H_2_O reactions^16–19^. Experimental measurement suggested that the day-time atmospheric lifetime of methanol in presence of OH radical is ~ 2 weeks^3^. Because of its long lifetime, effect of ammonia and water molecules on the OH + CH_3_OH reaction has been re-investigated and may be useful for the benchmark performance of atmospheric models. For that purpose, the details chemical kinetic investigation was done using ab initio/DFT methods coupled with statistical rate theories. The comparison of reaction energies and rate coefficients for OH + CH_3_OH, and in presence of ammonia/ water provide more confidence in our results.

## Theoretical methodology

### Quantum chemical calculations

All the electronic structure calculations were performed with the Gaussian 09 suite of programs^27^. Stationary points on the PES for OH + CH_3_OH, OH + CH_3_OH (+ NH_3_) and OH + CH_3_OH (+ H_2_O) reactions were computed using M06-2X/6-311++G(3df,3pd) level^28^ (see Table S1). To estimate the zero-point corrections (ZPE), normal-modes of vibrational frequency was performed at optimized geometries. The optimized transition states (TSs) have one imaginary frequency whereas reactants, complexes, and products have all positive vibrational frequencies. To improve the accuracy of energy, the single point energy calculations were performed at CCSD(T)/6-311++G(3df,3pd) level. As discussed in the previous work, the combination of CC and M06-2X with 6-311++G(3df,3pd) basis set typically gives results that are accurate to ~ 1–2 kcal/mol and is probably of the order of ~ 1 kcal mol^−1^ in the current study^16–19^. The spin expectation value < S^2^ > for each species was calculated and found in between ~ 0.75–0.85, indicating that spin contamination was negligible. For barrierless reaction pathways, ωB97XD/6-311++G(3df,3pd) were used to account for the empirical dispersion correction^29^. We have also performed the calculation using CBS-QB3 level and results are given in Supporting Information.

### Chemical kinetics calculations

The two steps of the radical–molecule reactions schemes can be expressed as

1a $${\text{R}} + {\text{X}}\mathop{\longrightarrow}\limits^{{{\text{k}}_{1} }}{\text{R}} \cdots {\text{X}}$$

1-a $${\text{R}} \cdots {\text{X}}\mathop{\longrightarrow}\limits^{{{\text{k}}-1}}{\text{R}} + {\text{ X}}$$

1b $${\text{R}} \cdots {\text{X}}\mathop{\longrightarrow}\limits^{{k_ {2} }}{\text{P}}$$

where *k*_1_ is the bimolecular forward rate coefficients (cm^3^ molecule^−1^ s^−1^) and *k*_–1_ is unimolecular reverse rate coefficients (s^−1^) and *k*_2_ is the unimolecular rate coefficient (s^−1^) for the second step.

According to Eq. 1, the net rate of the reaction of the complex R···X satisfies the following equation

2 $$\frac{d[\mathrm{R}\cdot \cdot \cdot \mathrm{X }]}{dt}={\mathrm{k}}_{1}\left[R\right]\left[X\right]-{\mathrm{k}}_{-1} \left[\mathrm{R}\cdot \cdot \cdot \mathrm{X}\right]-{\mathrm{k}}_{2}\left[\mathrm{R}\cdot \cdot \cdot \mathrm{X}\right]$$

At the steady state condition

3 $$\frac{d[\mathrm{R}\cdot \cdot \cdot \mathrm{X }]}{dt}={\mathrm{k}}_{1}\left[R\right]\left[X\right]-{\mathrm{k}}_{-1} \left[\mathrm{R}\cdot \cdot \cdot \mathrm{X}\right]-{\mathrm{k}}_{2}\left[\mathrm{R}\cdot \cdot \cdot \mathrm{X}\right]=0$$

The steady-state model leads to a rate coefficient for the overall reaction, which can be written

4 $${k}_{\mu \text{-CVT}}=\frac{{k}_{1}\times {k}_{2}}{{k}_{-1}+ {k}_{2}}$$

where *k*_*1*_ is microcanonical variational transition state theory ( $\mu {\text{VTST}}$) rate coefficients for OH + CH_3_OH → CH_3_OH···OH were calculated using Multiwell_ktools Program^30–32^, *k*_*-*1_ is $\mu {\text{VTST}}$ reverse rate coefficients obtained from equilibrium constant. The thermally $\mu {\text{VTST}}$ for CH_3_OH···OH → CH_3_OH + OH were calculated based on our previous works and details procedure are given in the references^30–32^. The *k*_*2*_ is $k_{CVT}^{unimol}$ unimolecular rate coefficients based on CVT/SCT approach were computed using PolyRate and GaussRate suite of programs^33,34^. The rate coefficients for CH_3_OH···OH → TSa → CH_3_O + H_2_O and CH_3_OH···OH → TS_b_ → CH_3_O + H_2_O were calculated using PolyRate and GaussRate suite of programs^33,34^.

The generalized rate coefficients were calculated by minimizing the transition state dividing surface along the reaction coordinate to get the canonical variational transition state theory (CVT) rate coefficients, which is given by Eq. (5) and Eq. (6):

5 $${k}^{GT}\left(T,s\right)=\Gamma {L}^{\ne }\times \frac{{k}_{B}T}{h}\frac{{Q}_{TS}^{\ne }(T,s)}{{Q}_{R}(T)}exp\left(-\frac{{V}_{MEP}(s)}{{k}_{B}T}\right)$$

6 $${k}^{CVT}\left(T\right)={min}_{s}{ k}^{GT}\left(T,s\right)={ k}^{GT}\left(T,{s}^{CVT}(T\right)$$

where ${k}^{GT}\left(T,s\right)$ and ${k}^{CVT}\left(T\right)$ are the rate coefficients of generalized and canonical variational,

transition state theory, respectively, *V*_*MEP*_ is the classical barrier height, $\Gamma$ is the small curvature tunneling (SCT) correction as implemented in Polyrate^33^, *h* is Planck’s constant, *k*_*B*_ is the Boltzmann constant, and ${Q}_{TS}^{\ne }$ and ${Q}_{R}$ are the total partition functions for the transition state and the reactants, respectively. The rate coefficients were calculated using a dual dynamic approach with CVT and the interpolated single point energies (ISPE) correction.

## Results and discussion

### Reaction pathways for OH + CH_3_OH

**Figure 1:**
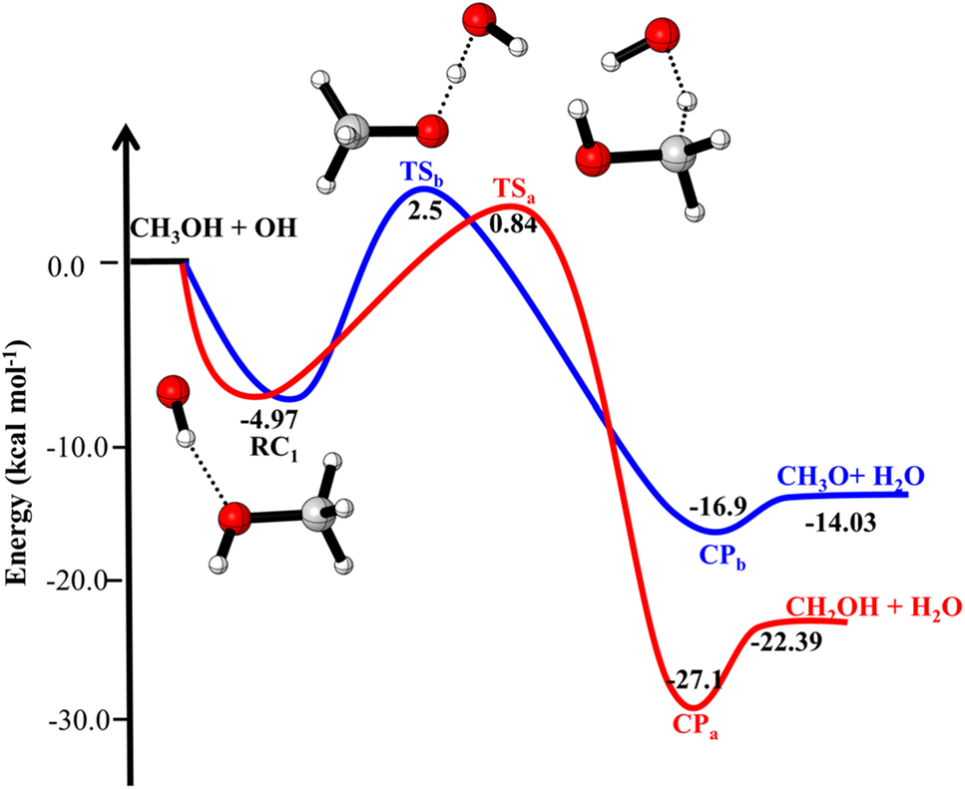
Zero-point corrected potential energy surface for OH + CH_3_OH reaction obtained using CC//M06-2X with 6-311++G(3df,3pd) basis set.

**Figure 2:**
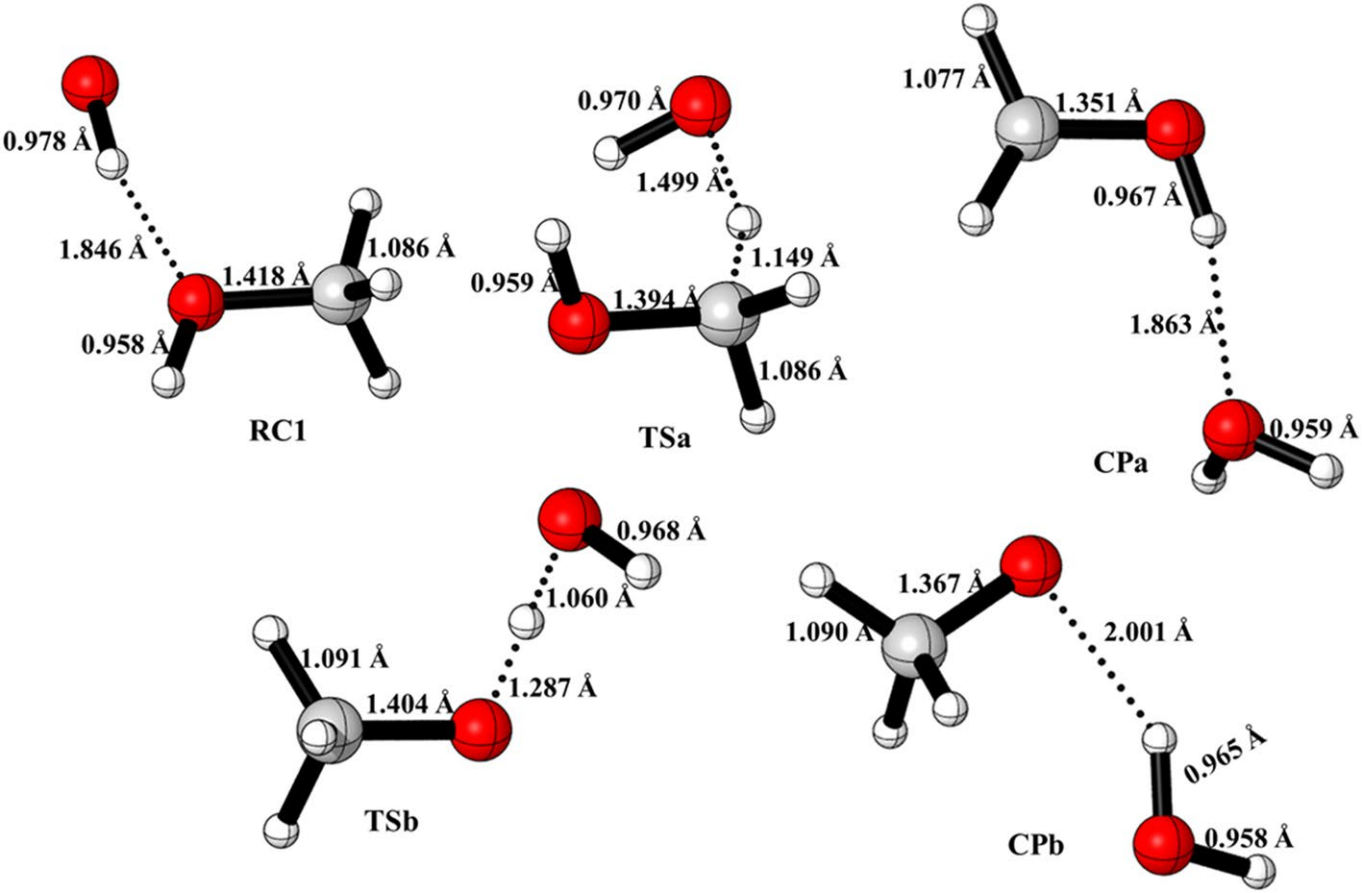
Structural and geometrical changes during OH + CH_3_OH reaction calculated using M06-2X/6-311++G(3df,3pd). These structures were generated from CYLview software.

The zero-point corrected PES for the water-free OH + CH_3_OH reaction is shown in Fig. 1. The energies of complexes, transition states (TSs) and products are shown relative to the energy of the reactants. The optimized structures of complexes and TSs are shown in Fig. 2. As shown in the figure, reaction channel a is more exothermic and has lower barrier heights (5.8 kcal/mol,) than channel b (7.5 kcal/mol); the former is therefore predicted to be both kinetically and thermodynamically more favorable.

The reaction proceeds via the formation of a pre-reactive complex (RC_1_) with C_1_ point group of symmetry in which the H of OH group is directed towards O of CH_3_OH. The energy of RC_1_ is 4.97 kcal mol^−1^ lower than the reactants and is in good agreement with the previous theoretical values^4,5,12^. To abstract the hydrogen atom from CH_3_ and OH group, OH of RC_1_ rotate in the plane until O atom come closer to the CH_3_ and OH group of CH_3_OH to pass via the geometry of TS_a_ and TS_b_. The barrier heights for hydrogen abstraction from CH_3_ group is 5.8 kcal/mol. This value is in good agreements with the value reported by Nguyen et al.^5^, Gao et al.^4^ and Jara et al.^12^ The barrier heights for H-abstraction reaction from OH group (~ 7.5 kcal/mol) is also in good agreement with previously reported values^4,5,12^.

#### Reaction pathways for OH + CH_3_OH (+ NH_3_)

In the presence of ammonia, the simultaneous collision of CH_3_OH, OH, and NH_3_ molecules are most unlikely, therefore, the reaction will occur through the formation of a two-body complex, and then this complex collides with a third species to form the three-body complexes. As discussed in our earlier studies^16,17^, we believe the formation of CH_3_OH···NH_3_ or OH···NH_3_ formed first, followed by an attack of the third molecule to these complexes. The complexes CH_3_OH ···NH_3_ and OH···NH_3_ are assumed to be more important than CH_3_OH···OH. This assumption is based on our previous studies on the similar reaction OH + CH_2_CH_2_ (+ H_2_O), OH + CH_2_O (+ H_2_O) and OH + CH_2_NH (+ H_2_O)^16,17^. Because the atmospheric concentration of CH_3_OH···OH is smaller than the OH···NH_3_ and CH_3_OH···NH_3_. Therefore, CH_3_OH···OH + NH_3_ is not considered in this work. In those two cases, most probable reaction pathways consist of two consecutive bimolecular elementary steps followed by unimolecular pathways:

7a $${\text{CH}}_{3} {\text{OH}} \cdots {\text{NH}}_{3} + {\text{ HO }} \to {\text{ CH}}_{2} {\text{OH }} + \, \left( {{\text{H}}_{2} {\text{O}}} \right) + {\text{ NH}}_{3}$$

7b $${\text{CH}}_{3} {\text{OH}} \cdots {\text{NH}}_{3} + {\text{ HO }} \to {\text{ CH}}_{3} {\text{O }} + \left( {{\text{H}}_{2} {\text{O}}} \right) + {\text{ NH}}_{3}$$

8a $${\text{CH}}_{3} {\text{OH}} + {\text{ NH}}_{3} \cdots {\text{HO}} \to {\text{ CH}}_{2} {\text{OH }} + \, \left( {{\text{H}}_{2} {\text{O}}} \right) + {\text{ NH}}_{3}$$

8b $${\text{CH}}_{3} {\text{OH}} + {\text{ NH}}_{3} \cdots {\text{HO}} \to {\text{ CH}}_{3} {\text{O }} + \, \left( {{\text{H}}_{2} {\text{O}}} \right) + {\text{ NH}}_{3}$$

**Figure 3:**
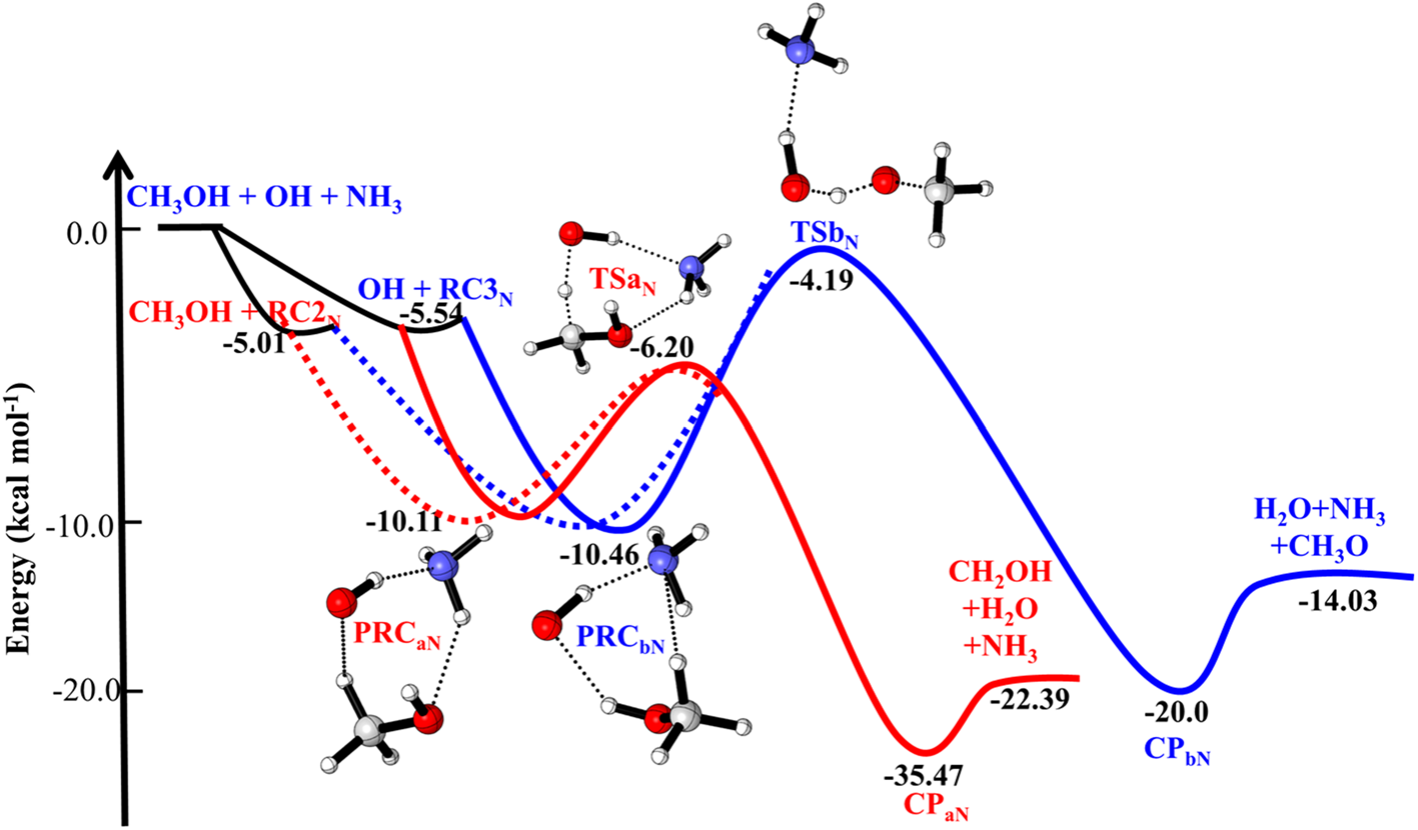
Potential energy surface for ammonia-assisted OH + CH_3_OH reaction obtained using CC//M06-2X with 6-311++G(3df,3pd) basis set. The relative energies include zero-point corrections.

**Figure 4:**
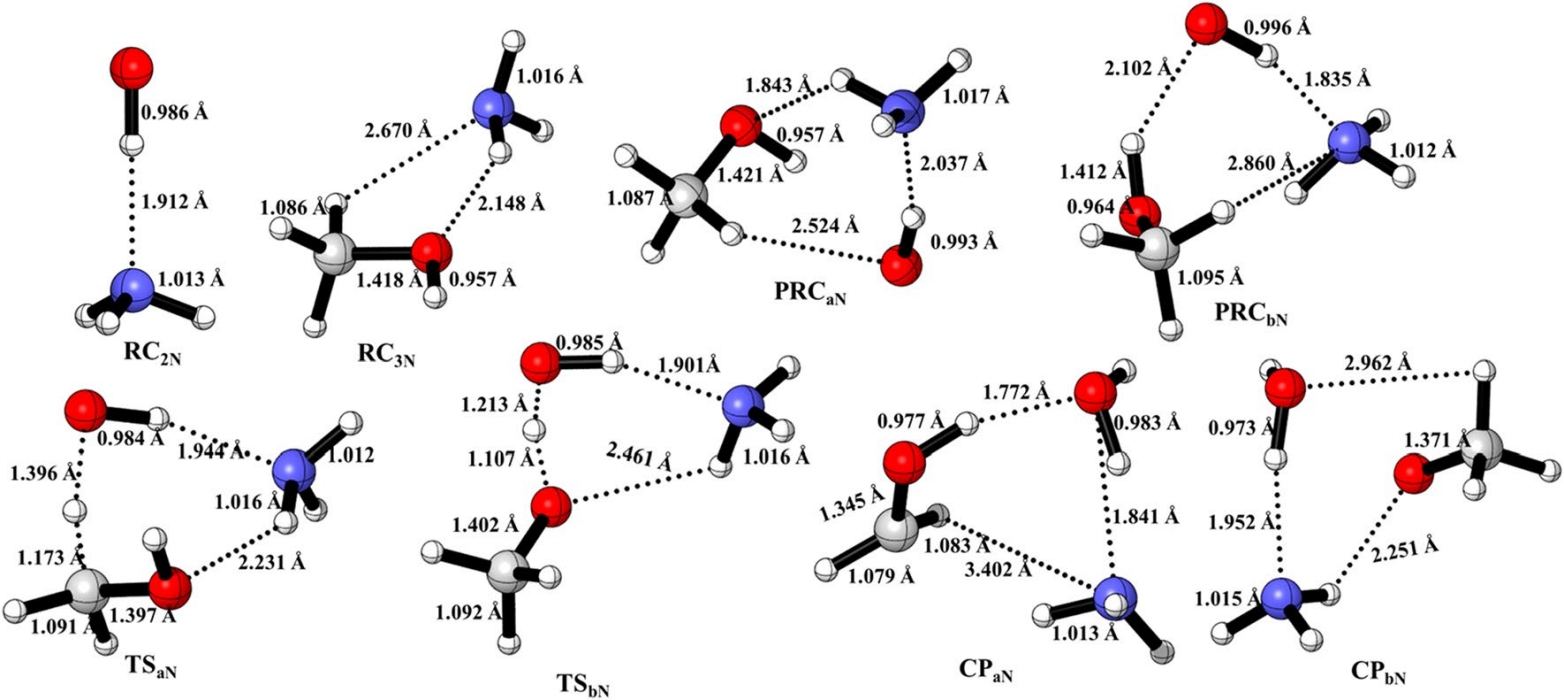
Optimized structures of ammonia-assisted pre-reactive complexes, transition states and post-reactive complexes were obtained using M06-2X/6-311++G(3df,3pd).

For ammonia-assisted reaction PES was carefully searched and all possible stationary points were identified. The zero-point corrected energies for ammonia-assisted OH + CH_3_OH reaction is shown in Fig. 3 and optimized structure of RCs, PRCs and TSs are shown in Fig. 4. The difference of binding energies between two-body complex CH_3_OH···NH_3_ (− 5.54 kcal/mol) and OH···NH_3_ (− 5.01 kcal/mol) is 0.5 kcal/mol. This BE is 1 kcal/mol higher than the value of OH···H_2_O^16^. This result is due to fact that the N–H hydrogen bond is stronger than O–H hydrogen bond. When CH_3_OH added to RC2_N_, and OH radical added to RC_3N_, five pre-reactive complexes were formed in which N of ammonia is acting as a proton donor and proton acceptor. Out of five PRC_N_, only two PRC_aN_ and PRC_bN_ are the most stable, therefore, other PRCs will not be considered in the kinetic calculations. As shown in Fig. 3, in both cases CH_3_OH + RC_2N_ and OH + RC_3N_ formed PRC_aN_ and PRC_bN_ with a BE of − 10.1 kcal mo1^–1^ and − 10.46 kcal/mol, respectively. The seven -membered ring structure of PRC_aN_ and its binding energy of − 10.1 kcal mol^–1^ are due to the combined effects of the N–H and O–H hydrogen bonds. The structure of these complexes is similar to the complexes found in water-assisted reaction^12,16^. The energies of these complexes are closer to the water-assisted complexes as discussed in earlier^12^ and later in “Reaction pathways for OH + CH_3_OH (+ H_2_O)” section. Starting from PRC_aN_, we have identified hydrogen abstraction by OH radical leading to CH_2_OH and CH_3_O. Transition state (TS1_aN_) corresponds to H abstraction reaction from methyl position. The calculated barrier height for this pathway (~ 4 kcal/mol), which is lower than the barrier height for OH + CH_3_OH (5.7 kcal/mol) reaction. The barrier heights of TS_bN_ is ~ 6.2 kcal/mol, which is ~ 1 kcal lower than the TS_bN_ of OH + CH_3_OH reaction. As suggested in OH + CH_3_OH reaction, the H-abstraction pathway from CH_3_ position is thermodynamically more favorable than the H- abstraction from OH bond. Addition of more ammonia *i.e.,* (NH_3_)_n>1_ may be further reduced the energies and become more thermodynamically favorable, but such reaction is not considered in the gas phase.

#### Reaction pathways for OH + CH_3_OH (+ H_2_O)

We have applied a similar approach for the water-assisted reactions as discussed in the case of ammonia and earlier studies^16–26^. Because the simultaneous collisions of OH, CH_3_OH and H_2_O, are very unlikely, the termolecular reaction probability is very small under true conditions either a CH_3_OH···H_2_O or OH···H_2_O is expected to form first, followed by an attack of the third molecule OH or CH_3_OH to this complex. In these two cases, most probable reaction pathways consist of two consecutive bimolecular elementary steps followed by unimolecular pathways:

9a $${\text{CH}}_{3} {\text{OH}} \cdots {\text{H}}_{2} {\text{O}} + {\text{ HO }} \to {\text{ CH}}_{2} {\text{OH }} + \, \left( {{\text{H}}_{2} {\text{O}}} \right)_{2}$$

9b $${\text{CH}}_{3} {\text{OH}} \cdots {\text{H}}_{2} {\text{O}} + {\text{ HO }} \to {\text{ CH}}_{3} {\text{O }} + \, \left( {{\text{H}}_{2} {\text{O}}} \right)_{2}$$

10a $${\text{CH}}_{3} {\text{OH}} + {\text{H}}_{2} {\text{O}} \cdots {\text{HO}} \to {\text{ CH}}_{2} {\text{OH }} + \, \left( {{\text{H}}_{2} {\text{O}}} \right)_{2}$$

10b $${\text{CH}}_{3} {\text{OH}} + {\text{H}}_{2} {\text{O}} \cdots {\text{HO}} \to {\text{ CH}}_{3} {\text{O }} + \, \left( {{\text{H}}_{2} {\text{O}}} \right)_{2}$$

**Figure 5:**
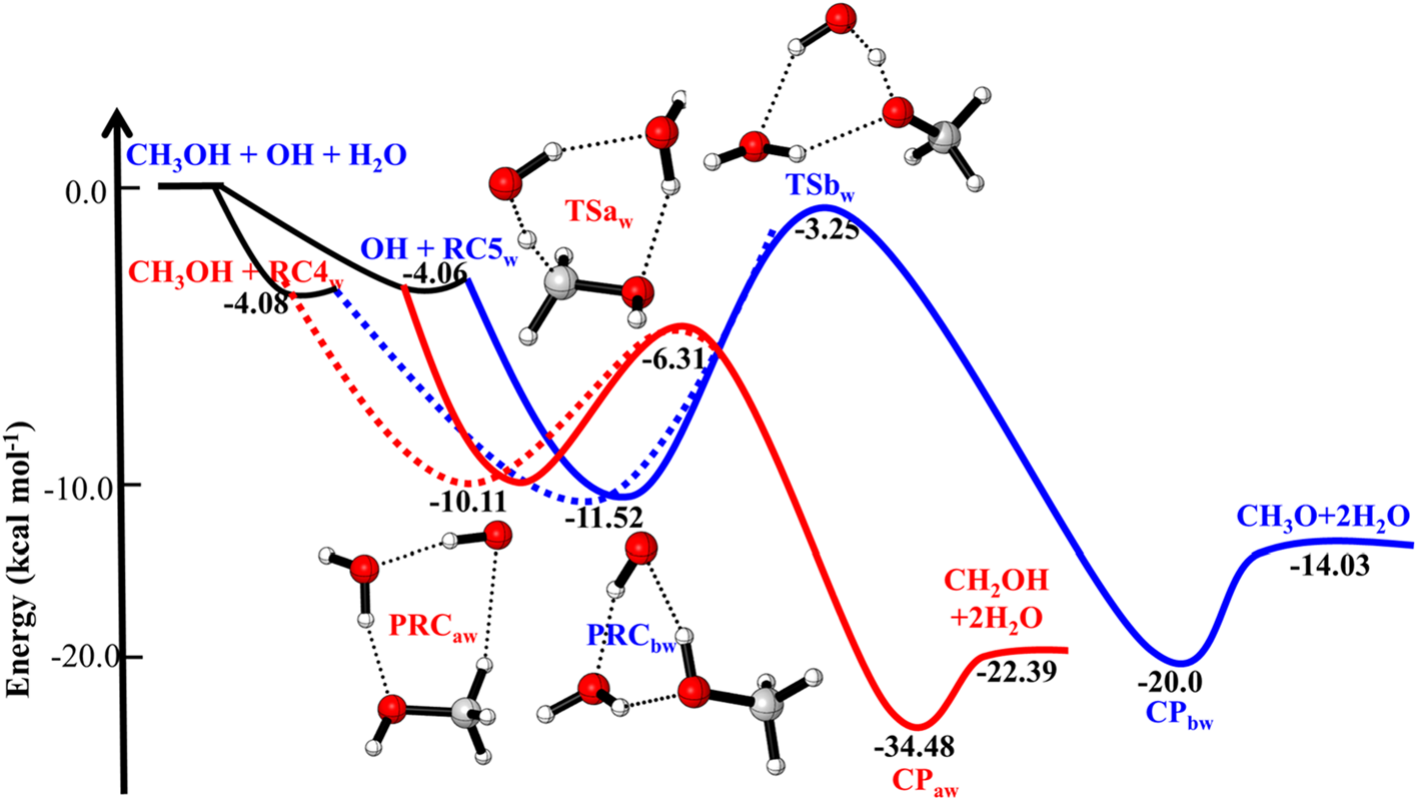
Potential energy surface for water assisted OH + CH_3_OH reaction obtained using CC//M06-2X with 6-311++G(3df,3pd) basis set. The relative energies include zero-point corrections.

**Figure 6:**
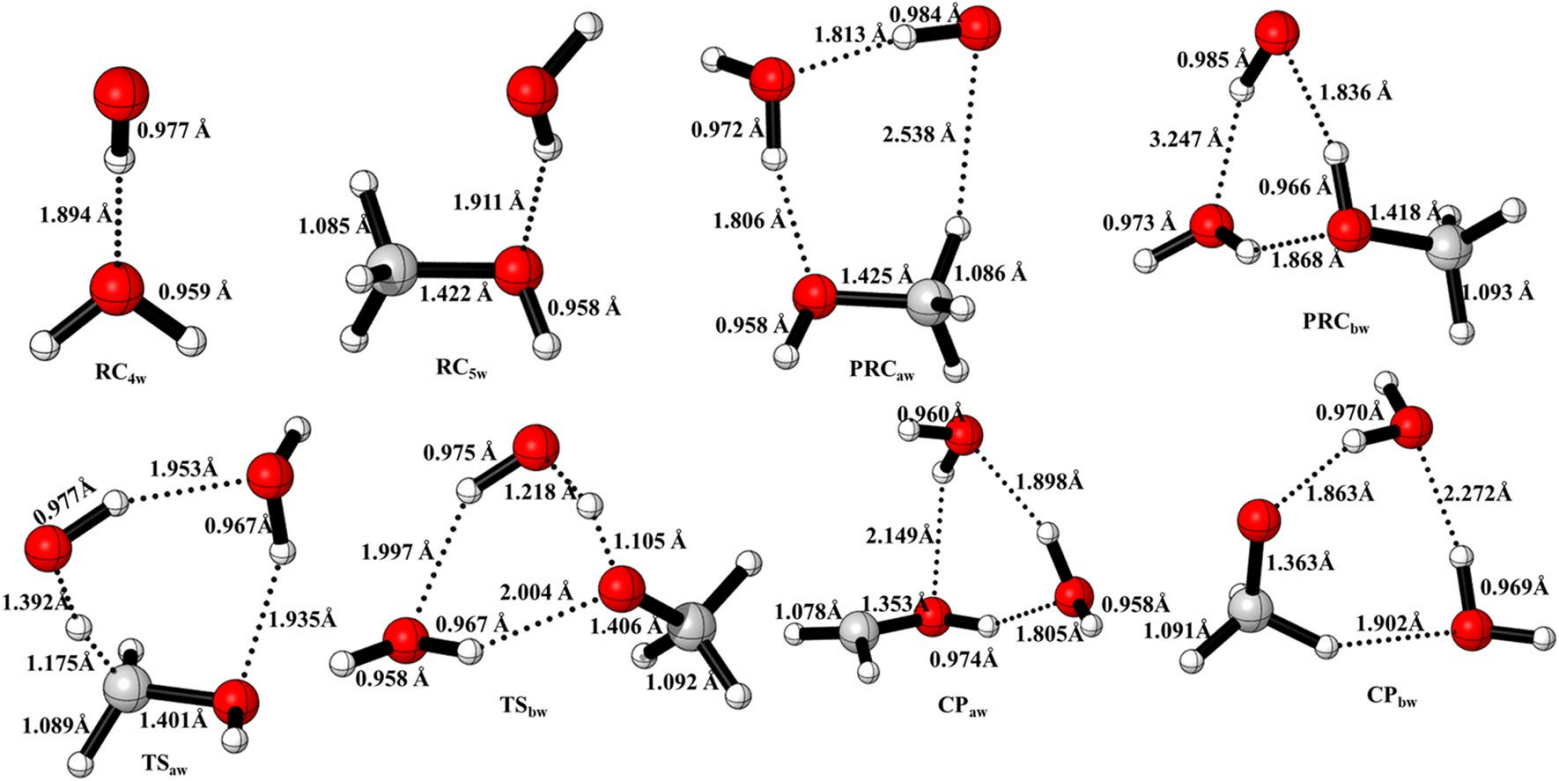
Structures of water-assisted pre-reactive complexes, transition states and post-reactive complexes were optimized using M06-2X/6-311++G(3df,3pd).

When a water molecule is added to the OH + CH_3_OH, the reaction proceeds via similar reaction channels *i.e.,* pathway a and pathway b but reaction mechanism becomes quite complex, yielding different isomers of pre-reactive complexes and transition states. As discussed in earlier studies^14^, we have also found different isomers of pre-reactive complex and transition states. The zero-point corrected energies of complexes, transition states, and products are shown in Fig. 5 using CCSD(T)//M06-2X/with 6-311++G(3df,3pd) basis set. The optimized structure of complexes and TSs are shown in Fig. 6. We have found multiple structures of transition states and pre-reactive complex. For the simplicity, we have used only the minimum energy structure as discussed in earlier work^12,14^.

The calculated binding energy (BE) between CH_3_OH and H_2_O (− 4.06 kcal/mol) is in very good agreement with value (− 4 kcal/mol) reported previously^12,14^. The BE of H_2_O···OH (− 4.0 kcal/mol) is nearly same the BE of CH_3_OH···H_2_O (− 4.0 kcal/mol). This is due to fact that the hydrogen bond (O···H) in H_2_O···OH is very similar to the C–O···H interaction in CH_3_OH···H_2_O.

When a third molecule (CH_3_OH or OH radical) attacked to these complexes *i.e.,* H_2_O···OH, CH_3_OH···H_2_O, different pre-reactive complexes isomers were observed. Out of these PRC_w_, we have chosen only most stable PRCs, *i.e.*, PRC_aw_ and PRC_bw_ in the current study. The BE of PRC_aw_ (− 10.8 kcal/mol) is the combined effect of O···H and C···H interactions. Starting from PRC_aw_, we have identified one reaction pathway, *i.e.*, hydrogen abstraction by OH radical on methyl carbon. Transition state (TS1_aw_) corresponds to H abstraction reaction from methyl position. The calculated barrier height for this pathway (~ 4 kcal/mol), which is lower than the barrier height for water-free reaction (~ 6 kcal/mol). The hydrogen abstraction from O–H bond leads to form a product via transition state (TS_bw_) correspond to H-abstraction reaction. The barrier height of TS2_bw_ (~ 6 kcal/mol) is lower than value the value of TS2_b_ (~ 7.5 kcal/mol). The PRC_bw_ has different structure than PRC_aw_ and energetically 1 kcal/mol higher.

### Enthalpies of reactions

The enthalpies relative to the reactants for OH + CH_3_OH, and ammonia/water assisted reaction are given in Table 1. This table also includes enthalpies values from the literature values^5,12,14,35–37^. The enthalpies of reaction of OH + CH_3_OH → CH_2_OH + H_2_O and OH + CH_3_OH → CH_3_O + H_2_O agree with the ATcT thermochemical data base^35–37^ within ~ 1 kcal/mol, respectively and are in excellent agreement with previous theoretical calculations^12^. As shown in Fig. 4, the formation of the complex in OH···CH_3_OH···NH_3_ (PRC_aN_) and OH··· CH_3_OH ···NH_3_ (PRC_bN_), structurally and energetically they are different (see Fig. 4 and Table 1). The different BE of PRC_aN_ (− 10.1 kca/mol) and PRC_bN_ (− 10.5 kcal/mol) is due to the different orientation of OH and NH_3_ molecules.

**Table1:** Enthalpies of reaction (∆*H*_*rxn*_ (0 K) in kcal mol^−1^ of OH + CH_3_OH reaction.

OH + CH_3_OH →	This work	Literature^a,b,c^
OH···CH_3_OH (RC1)	− 4.97	− 4.37, − 4.47, 4.90, 4.82^a^
HO···HCH_2_OH (TS_a_)	− 0.84	0.1, 0.36^a^
HO···HOCH_3_ (TS_b_)	2.50	1.91, 3.29^a^
CH_2_OH + H_2_O	− 22.39	− 23.13, 23.1, 23.09^b^
CH_3_O + H_2_O	− 14.03	− 13.66, 13.73^b^

OH + CH_3_OH + NH_3_ →	This work	Literature
OH···CH_3_OH···NH_3_ (PRC_aN_)	− 10.11	–
OH···CH_3_OH···NH_3_(PRC_bN_)	− 10.45	–
OH··· HCH_2_OH ···NH_3_ (TS_aN_)	− 6.2	–
OH···HO···HOCH_3_···NH_3_ (TS_bw_)	− 4.19	–
CH_2_OH + H_2_O + NH_3_	− 22.39	–
CH_3_O + H_2_O + NH_3_	− 14.03	

OH + CH_3_OH + H_2_O →	This work	Literature^a^
OH···CH_3_OH···H_2_O (PRC_aw_)	− 10.80	− 10.80^c^
OH··· CH_3_OH···H_2_O (PRC_bw_)	− 11.52	− 12.03^c^
OH··· HCH_2_OH···H_2_O (TS_aw_)	− 6.31	− 4.98^c^
OH···HO···HOCH_3_···H_2_O (TS_bw_)	− 3.25	− 2.74^c^
CH_2_OH + 2H_2_O	− 22.39	− 23.09^b^
CH_3_O + 2H_2_O	− 14.03	− 13.73^b^

^a^Nguynet al.^5^, ^b^AT_C_T^35–37^, ^c^Jara et al.^12^.

The calculated BE of PRC_aw_ (− 10.1 kcal/mol) and PRC_bw_ (− 11.5 kcal/mol) are in very good agreements with the value (− 9.3 kcal/mol) reported by Jara-Toro et al.^12^ The energies of PRCs and transition states of reaction systems were also compared with the theoretically available value ( ) and products energies were compared with the most accurate set of experimental value from Active Thermochemical Tables. The PRC_aN_ and (PRC_bN_), are structurally similar to those of PRC_aw_ and PRC_bw_. The energy difference between these complexes is due to the presence of N–H bond.

On the basis of the energetics summarized in Table 1, it is clear that barrier height for the abstraction from CH_3_ group is small compared to abstraction from OH group in all the cases cussed here. The effect of ammonia and water leads to the lower barrier height and makes the reaction more thermodynamically favorable.

As suggested by the reviewer, we have used another level of quantum chemical method to justify the accuracy of the calculations. We have re-done the calculation using CBS-QB3 level as suggested by the reviewer. The results are given in supporting information Table S5 and rate coefficient plots are shown in Supporting Information Figure S4 and Figure S5. The calculated energies for both the transition state is 0.5 kcal/mol difference than the CC/M06 level and rate coefficients for CH_3_OH + OH reaction is nearly factor of 5 higher than the experimental measurement. Therefore, we believe the results of CBS-QB3 did not improve as we expected. As suggested in Nguyen et al.^5^ study, the TSs of CH_3_OH + OH are very sensitive to the quantum chemical methods, even they used HEAT Protocol for the energies calculation, and their results are off by factor of 2 from the experimental measurement. Nguyen et al.^5^ also adjusted their barrier heights to get accurate results. In our earlier work^38^ on CH_2_NH + OH, we used CCSDT/aug-cc-pvtz for optimization and energies calculation, due to sensitive nature of barrier height, we adjusted the barrier height by 0.3 kcal/mol, and adjusted value agreed with the experimentally measure value. This adjustment is well within the estimated accuracy of the theoretical methods (~ 2 kcal/mol) as suggested in the earlier studies^38^.

### Rate coefficients

#### OH + CH_3_OH reaction

**Figure 7:**
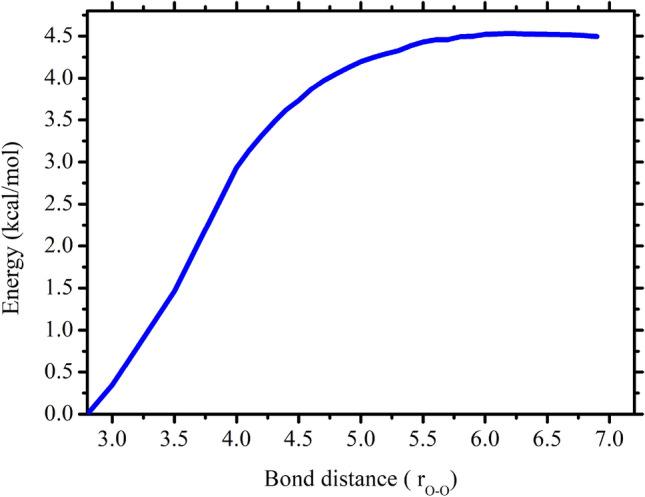
Zero-point corrected potential energy profile dissociation of the CH_3_OH···HO as functions of R_O–O_ distances computed at the CCSD(T)/6-311++G (3df,3pd)//ωB97XD/6-311++G(3df,3pd) level.

To the best of our knowledge until now, the details of interaction between OH and CH_3_OH in the temperature range of 200–400 K had not been investigated, despite the large rate coefficient recommended in the literature for OH + CH_3_OH leading to CH_3_O/CH_2_OH + H_2_O^4,5,12,14^. The CH_3_OH···HO that assume to play important roles in the HO + CH_3_OH reaction system is formed via the entrance channels which have the well depth of ~ 5 kcal/mol. Figure 7 shows the zero-point corrected potential energy for the entrance channel forming CH_3_OH···HO.

**Figure 8:**
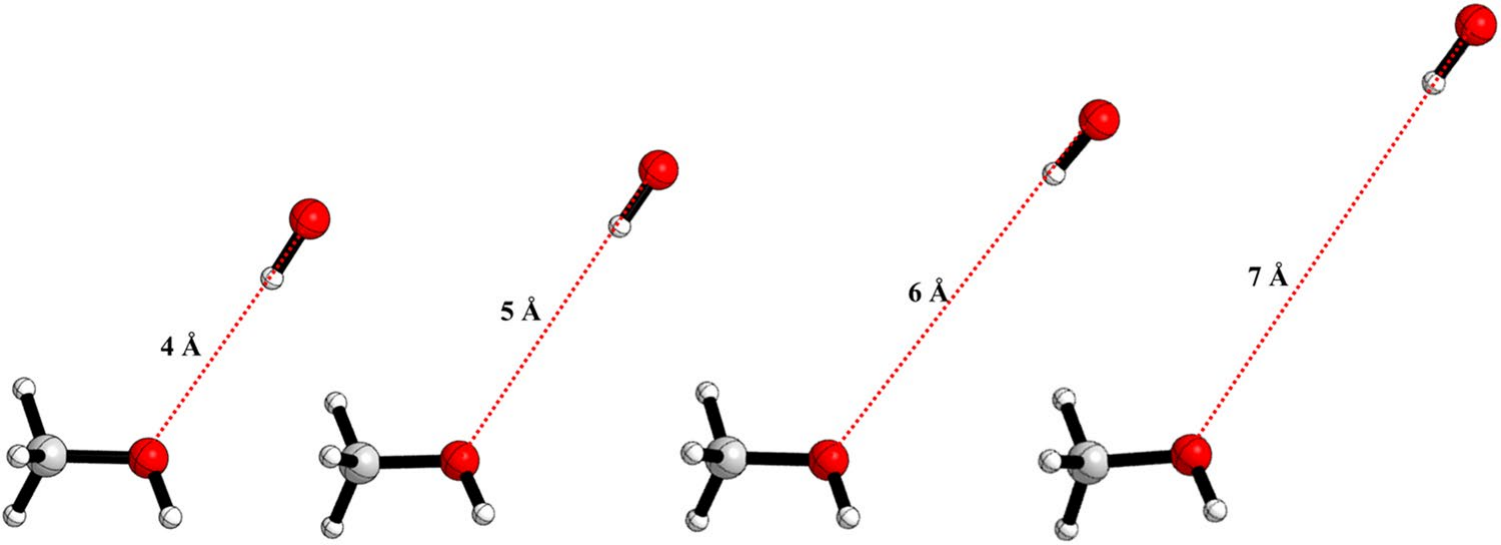
Dissociation of CH_3_OH···OH at several R_O−O_ distances along the reaction of OH + CH_3_OH.

To locate the transition state of dissociation of CH_3_OH···HO, the potential energies (including zero-point energies) were computed in a series of constrained optimizations as a function of the R_O-O_ bond distance (from 3 to 7 Å). The optimized geometries at some points along with the reaction pathways are shown in Fig. 8.

The CVTST "trial" rate coefficients were computed in the temperature range of 200–400 K along the reaction path is shown in Supporting Information Figure S1. At each temperature, the plot shows a single minimum between 5.5 and 6.0 Å. The rate coefficients for dissociation and association reaction were computed using microcanonical approaches and values are tabulated in Table 2.

**Table 2:** Calculated rate coefficients for the OH + CH_3_OH → CH_2_OH/CH_3_O + H_2_O calculated at CC/M06 level.

Temp	*k*_*1*_	*k*_*-1*_	*k*_*2-a*_	*k*_*2-b*_	${k}_{\mu -\mathrm{CVT}}$ (this work)^a^	Exp.value^2^
200	1.16 × 10^−11^	2.01 × 10^8^	6.72 × 10^5^	9.58 × 10^5^	3.50 × 10^−13^	5.70 × 10^−13^
225	1.23 × 10^−11^	7.78 × 10^8^	2.51 × 10^6^	3.01 × 10^5^	3.60 × 10^−13^	6.40 × 10^−13^
250	1.29 × 10^−11^	2.29 × 10^9^	7.79 × 10^6^	2.57 × 10^6^	4.10 × 10^−13^	7.22 × 10^−13^
275	1.36 × 10^−11^	5.50 × 10^9^	2.03 × 10^7^	6.82 × 10^6^	4.68 × 10^−13^	8.13 × 10^−13^
300	1.43 × 10^−11^	1.14 × 10^10^	4.63 × 10^7^	1.62 × 10^6^	5.42 × 10^−13^	9.15 × 10^−13^
325	1.50 × 10^−11^	2.09 × 10^10^	9.09 × 10^7^	3.47 × 10^7^	6.07 × 10^−13^	1.03 × 10^−12^
350	1.57 × 10^−11^	3.52 × 10^10^	1.62 × 10^8^	6.81 × 10^7^	6.76 × 10^−13^	1.15 × 10^−12^
375	1.64 × 10^−11^	5.51 × 10^10^	2.67 × 10^8^	1.24 × 10^8^	7.48 × 10^−13^	1.28 × 10^−12^
400	1.72 × 10^−11^	8.15 × 10^10^	4.13 × 10^8^	2.11 × 10^8^	8.24 × 10^−13^	1.42 × 10^−12^
k = AT^n^ exp(− B/T)	A = 5.09 × 10^−14^ n = 0.93 B = − 101.52	A = 1.62 × 10^15^ n = − 0.58 B = 2566.5	A = 1.53 × 10^7^ n = 1.47 B = 2188.4	A = 4.7 × 10^−40^ n = 17.28 B = − 2536.4	A = 1.01 × 10^−20^ n = 2.86 B = − 431.03	A = 3.82 × 10^−19^ n = 2.4 B = 300

k_1_ (cm^3^ molecule^−1^ s^−1^) is microcanonical VTST rate coefficient, k_-1_ (s^−1^) microcanonical reverse rate coefficient, k_2-a_ (s^−1^) is ${k}^{CVT}\left(T\right)$ rate coefficient for pathway a, k_2-b_ (s^−1^) is ${k}^{CVT}\left(T\right)$ rate coefficient for pathway b, ${k}_{\mu -\mathrm{CVT}}$ is total rate coefficient without adjustment (cm^3^ molecule^−1^ s^−1^).

The rate coefficients for the formation of CH_3_OH···HO is almost independent of temperature. The calculated value is also compared with similar type of reaction system and the values were very close to each other^16,26^. The calculated lifetime of the complex at 225 K, which is near to the altitude of 10–12 km is ~ 1 ns. This lifetime of the complex is too short to undergoes secondary reaction. In fact, 1 ns is very rapid, which could be negligible in the CH_3_OH + OH reaction and direct abstraction reaction could lead the products^5^. To the best of our knowledge the microcanonical VTST calculation for the forming the CH_3_OH···HO in the temperature range of 200–400 K is not known and were not discussed in earlier studies. As suggested in earlier studies^5^ that at high-pressure limit complex CH_3_OH···HO will completely be stabilized by the collisions with another atmospheric molecule and will be rapidly re-populated thermally. Once it re-populated, the complex decomposes to give the CH_2_OH + H_2_O and CH_3_O + H_2_O. We believe that rate coefficients calculation for the barrierless reaction using microVTST is better choice than the use of equilibrium approach for calculation of the total rate coefficient (see Eq. 3), which can reduce the error in the kinetic calculation at least by factor of 2.

**Figure 9:**
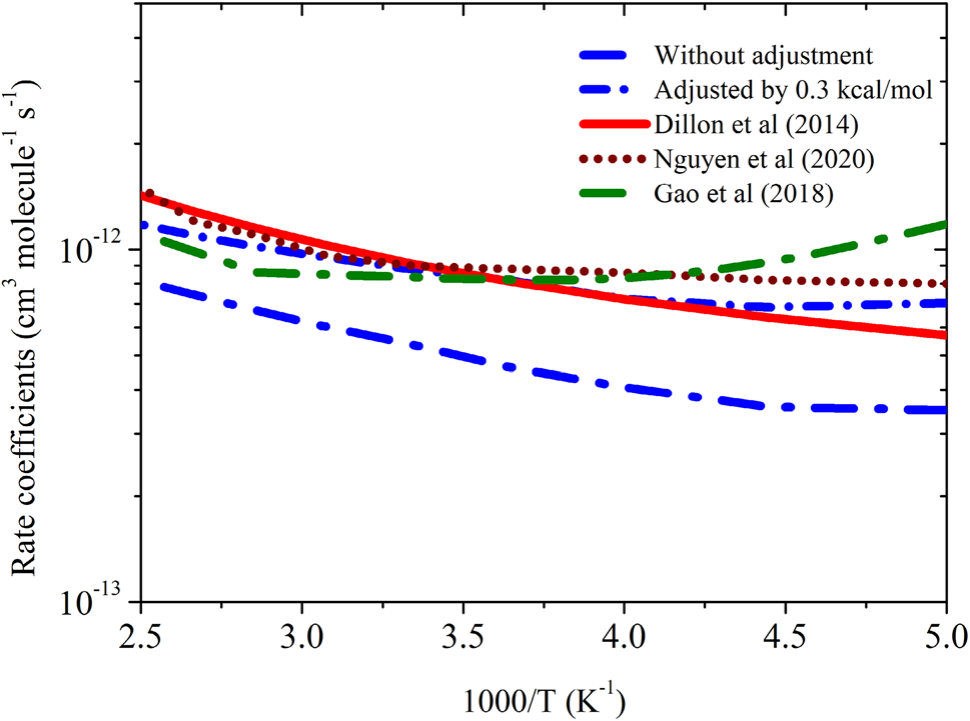
Rate coefficients for OH + CH_3_OH reaction.

The rate coefficients calculated using CC//M06-2X are shown in Fig. 9. Our Calculated value at 300 K (5.42 × 10^–13^ cm^3^ molecule^−1^ s^−1^) is in good agreement with the experimentally measured^2^ value (9.15 × 10^–13^ cm^3^ molecule^−1^ s^−1^) and theoretically^4,5^ calculated ones (6.2 × 10^–13^, 8.2 × 10^–13^ and 9.13 × 10^–13^ cm^3^ molecule^−1^ s^−1^)^4,5,12^. The calculated value is factor of ~ 2 lower than experimental values over the entire temperature range. This level of accuracy is sufficient for the present purposes, considering the expected errors in the computed thermochemistry. In the recent work Nguyen et al.^5^ suggested that the barrier heights of OH + CH_3_OH reaction is very sensitive to the quantum chemical calculation. They suggested that it is very challenging to calculate accurate energy using ab initio method for OH + CH_3_OH reaction even using HEATs protocol^5^. To match the calculated value with experimentally measured values, they adjusted the barrier heights by 0.4 kcal/mol^5^. We have also applied same approach and adjusted the barrier height of TS_a_ and TS_b_ by -0.3 kcal/mol. This adjustment is well within the estimated accuracy of the theoretical methods (< 1 kcal/mol), which brings the computed rate constants into almost exact agreement with the experimental values over 200–400 K. Similar adjustment was done in earlier research work to correct the reaction energies^38^. We have also calculated the percentage contribution of Pathway a (CH_2_OH + H_2_O, > 95%) and for Pathway b (CH_3_O + OH, < 5% in the temperature range of 200–400 K. This result is in very good agreement with the experimentally measured percentage contribution in the same temperature range^3^.

### Ammonia-assisted OH + CH_3_OH reaction

As discussed in “Reaction pathways for OH + CH_3_OH (+ H_2_O)” section and given in Eq. (7) and (8), only two entry channels CH_3_OH···NH_3_ + HO (Pathway A_n_) and CH_3_OH + NH_3_··· HO (Pathway B_n_) are considered for the rate coefficient calculations.
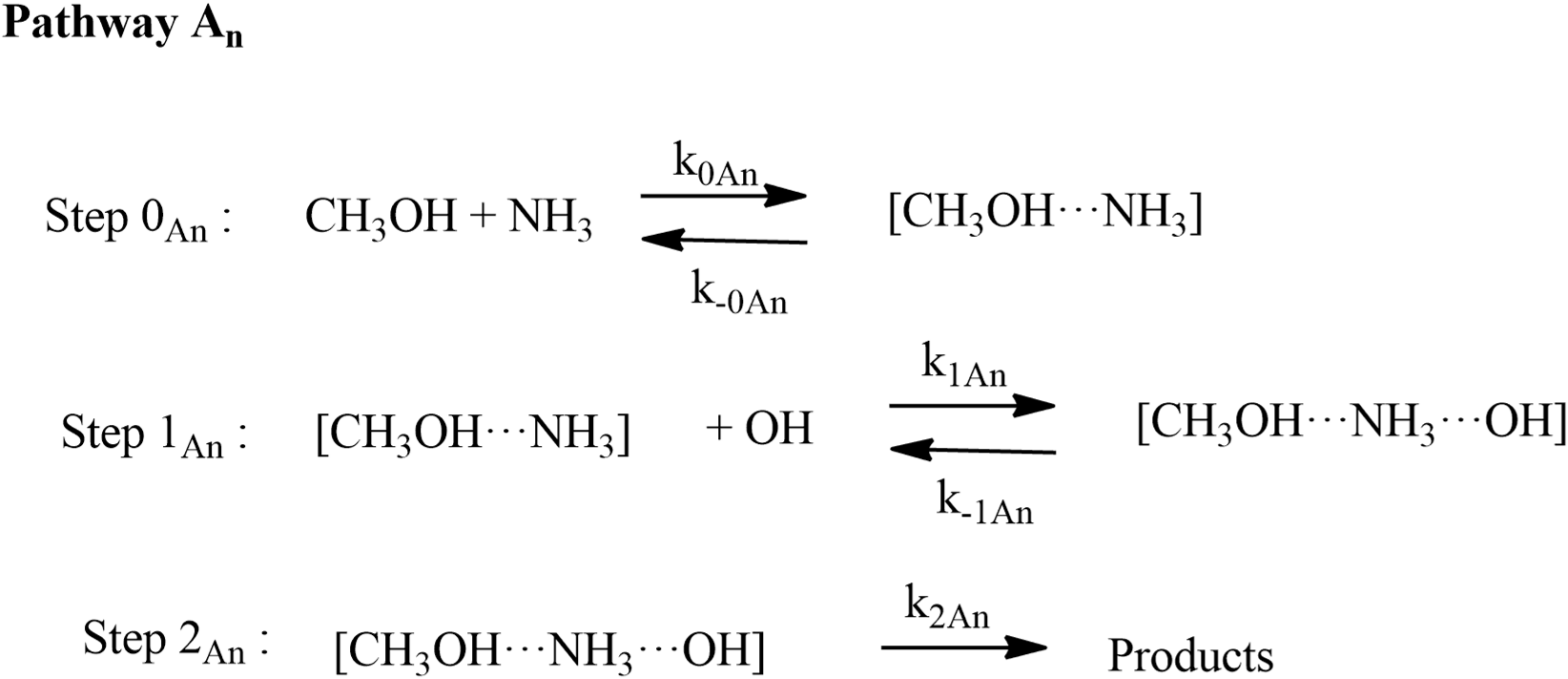

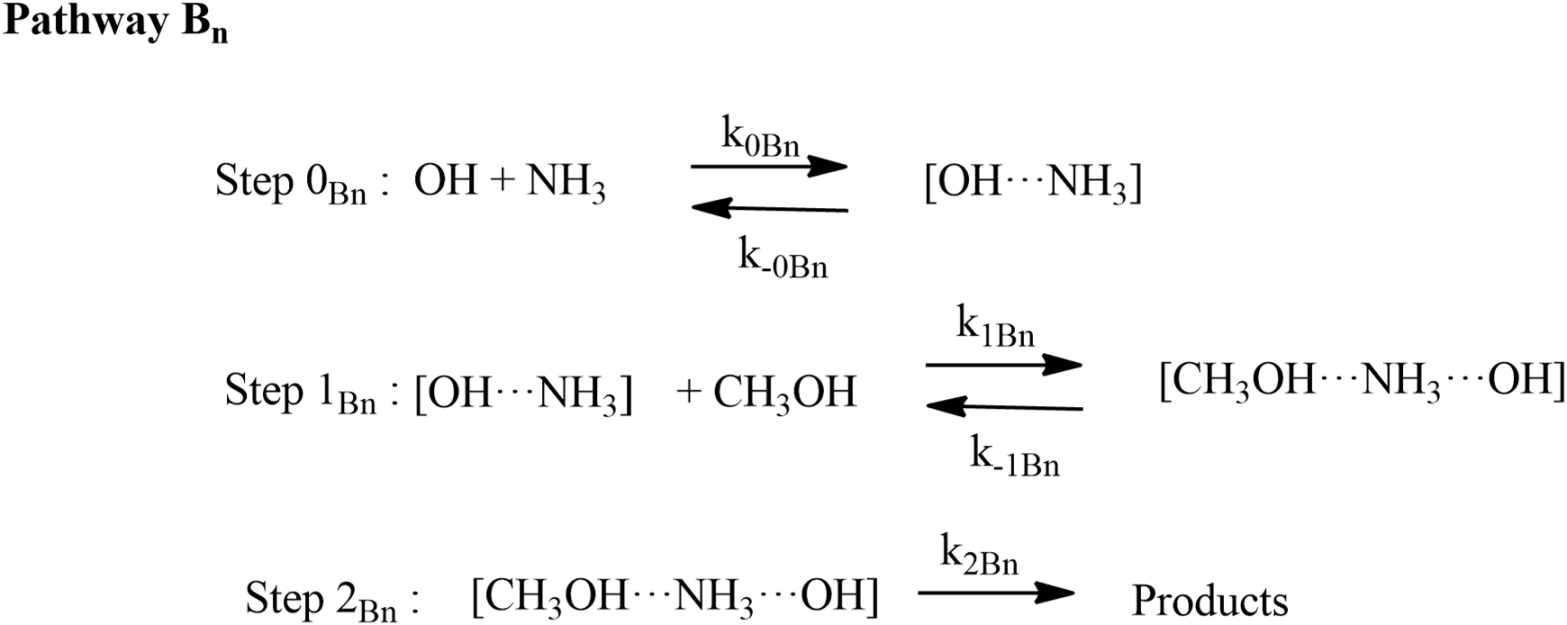


The rate coefficients for the reactions of OH + CH_3_OH (+ NH_3_) were calculated based on high-pressure limit condition. Because of the presence of two or more hydrogen bonds in PRC_N_, locating the TS of backward reaction *i.e.,* PRC_N_ → CH_3_OH···NH_3_ + OH and PRCs → CH_3_OH + NH_3_···OH using constrained optimization technique is more complicated than locating the TS for CH_3_OH···HO → OH + CH_3_OH, the latter has a single hydrogen bond. In that case, we have used the equilibrium approach to account for the presence of forward and backward reactions. The total rate coefficients in Eq. 4:${ k}_{\mu \text{-CVT}}=\frac{{k}_{1}\times {k}_{2}}{{k}_{-1}+ {k}_{2}}$ were assumed that k_-1_ >  > *k*_*2*_. $k = K_{eq} k_{2}$. This kinetic model is reasonably correct at the high-pressure limit and for the three-body complex, where the pre-reactive complex can be stabilized by collisions with other atmospheric species^18–25^.

The unimolecular rate coefficients (*k*_*2*_) were computed using CVT/SCT method and the rate coefficient (*k*_*2*_) values are tabulated in supporting information, Table S2. The equilibrium constants ($K_{eq}$) for the formation of two-body and three-body complexes were calculated based and the results are tabulated in Table S3 and Table S4.

The rate coefficients for HO + CH_3_OH (+ NH_3_) were calculated in the temperature range 200 to 400 K using CVT/SCT methods are shown in Fig. 9. The rate coefficients of Pathway A_*n*_ and Pathway B_*n*_ were calculated using the approach given in Eq. 11:

11 $${k}_{AN}^{CVT}={k}_{1N}+{k}_{2N} \mathrm{and} {k}_{BN}^{CVT}={k}_{{1}^{^{\prime}}}+{k}_{{2}^{^{\prime}}}.$$

${k}_{1N}={K}_{eq(E)}\times {k}_{TSaN}$, ${k}_{2n}={K}_{eq(G)}\times {k}_{TS{aN}}$, ${k}_{{1}^{^{\prime}}}={K}_{eq(F)}\times {k}_{TSbN}$, ${k}_{{2}^{^{\prime}}}={K}_{eq(H)}\times {k}_{TSbN}$ and are bimolecular rate coefficients of each reaction pathway involved in the OH + CH_3_OH reaction. The total rate coefficients for OH + CH_3_OH (+ NH_3_) are expressed by Eq. 12:

12 $${k}_{total-N}={k}_{AN}^{CVT}+{k}_{BN}^{CVT}$$

**Figure 10:**
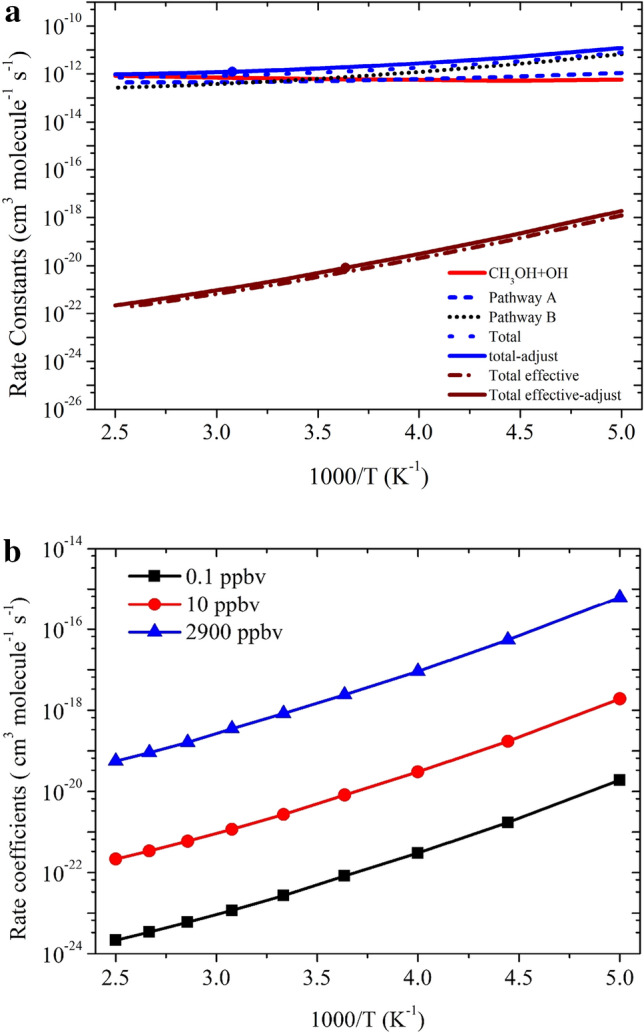
Rate coefficients for CH_3_OH + OH and CH_3_OH + OH (+ NH_3_) reactions at (**a**) 10 ppbv, (**b**) 0.1, 10 and 2900 ppbv.

The total rate coefficients in the temperature range of 200–400 K of Pathway A_*N*_ and Pathway B_*N*_ are also shown in Fig. 10. Our results show that the rate coefficients of Pathway B_*N*_ are higher than Pathway A_*N*_. In general, the rate coefficients of ammonia-assisted reaction is higher than the OH + CH_3_OH reaction in the temperature range of 200–400 K (see Fig. 10). Our calculations suggest that the catalytic effect takes place if step 0 is not included in the reaction mechanism. Ignoring step 0 is equivalent to assuming that all the methanol is complexed with ammonia, which is not true.

The correct expression to calculate the total effective rate coefficients by Eq. 13:

13 $${k}_{total-N}^{eff}=\left[\left\{{K}_{eq\left(5\right)}\times {k}_{AN}^{CVT}\right\}+\left\{{K}_{eq\left(4\right)}\times {k}_{BN}^{CVT}\right\}\right]\times \left[{NH}_{3}\right].$$

where ${K}_{eq(5)}$ and $K_{eq(4)}$ are equilibrium constants of CH_3_OH + NH_3_ → RC5, and NH_3_ + HO → RC_4_, reactions, respectively (see Table 2) and [NH_3_] is ammonia concentration used at 10 ppbv based on previous studies^6,8^.

The total effective rate coefficient for OH + CH_3_OH (+ NH_3_) (2.7 × 10^–21^ cm^3^ molecule^−1^ s^−1^ at 300 K) is ~ 8 order magnitude lower than OH + CH_3_OH reaction (8.8 × 10^–13^ cm^3^ molecule^−1^ s^−1^). This result is due to fact that the ammonia-assisted pathway depends on ammonia concentration (see Table 2). As discussed in dry OH + CH_3_OH reaction, without adjustment, the agreement with experiments was good, but adjustment gave in excellent agreement with the experimental data. Therefore, we adjusted the barrier height of the PRCA_n_ → TSA_n_ and PRCB_n_ → TSB_n_ channel by − 0.3 kcal/mol. The calculated rate coefficients are nearly same even after the adjustment of the (see Fig. 10) and show negative temperature-dependent, such behavior has also been observed for similar reactions system in the literature^7,8^. The rate coefficient at different NH_3_ concentration is shown in Fig. 10b. The calculated values are consistent with the previous studies of similar type of reaction^8^.

### Water-assisted OH + CH_3_OH reaction

The reaction mechanisms for OH + CH_3_OH in presence of a single water molecule are presented. To get more confidence in the predicted rate coefficients, we compared our results with previously published results^12,14^.
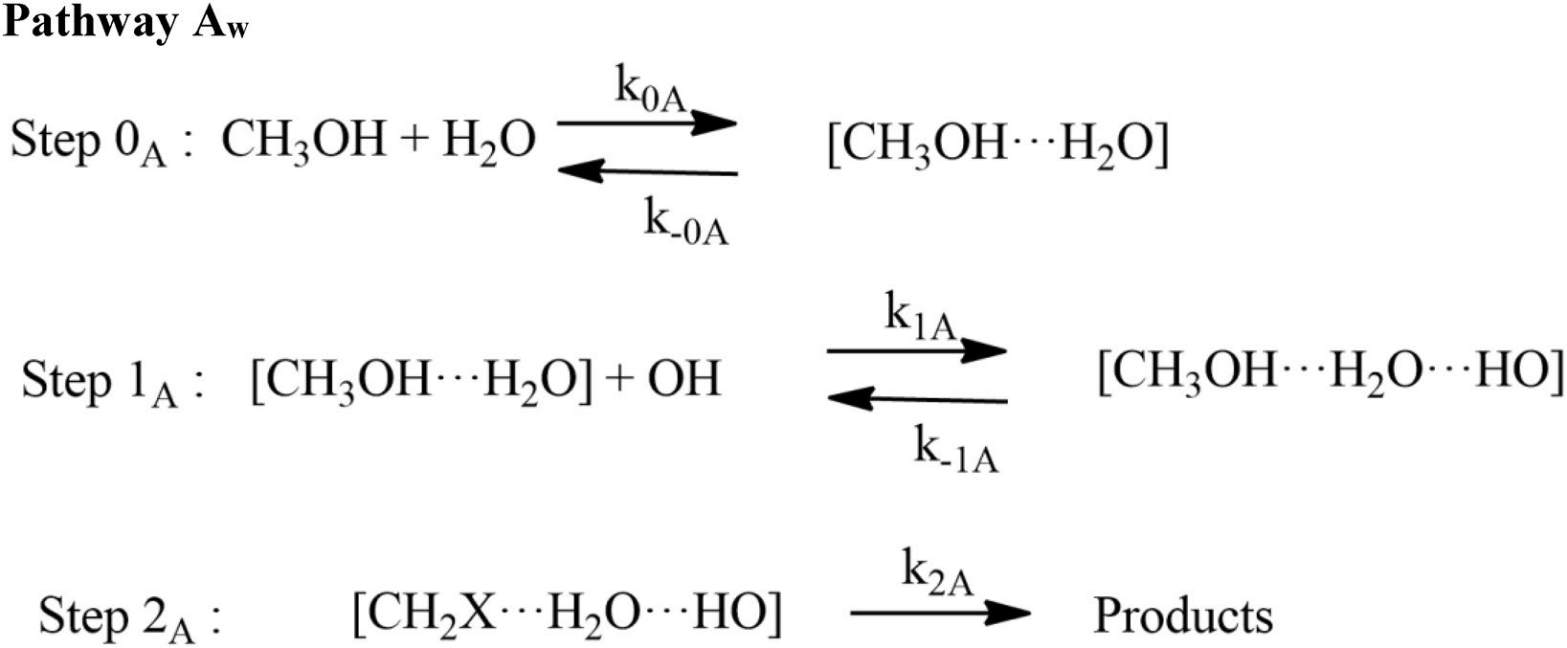

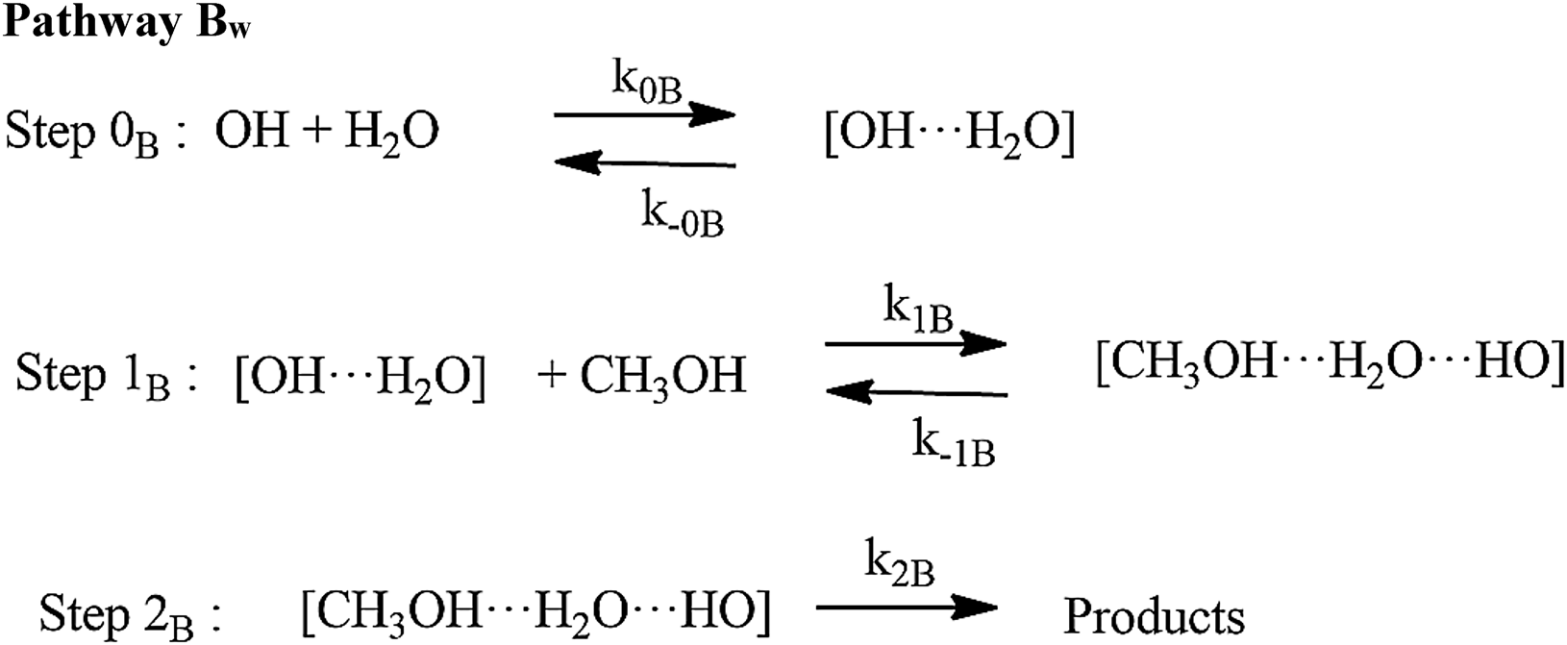


The rate coefficients for HO + CH_3_OH (+ H_2_O) were calculated in the temperature range 200 to 400 K using CVT/SCT methods (see Table S2). The rate coefficients of Pathway A_*w*_ and Pathway B_*w*_ were calculated using the approach given in Eq. 14

14 $${k}_{Aw}^{CVT}={k}_{3}+{k}_{4} \mathrm{and} {k}_{Bw}^{CVT}={k}_{{3}^{^{\prime}}}+{k}_{{4}^{^{\prime}}}.$$

where ${k}_{3}={K}_{eq(A)}\times {k}_{TSaw}$, ${k}_{4}={K}_{eq(C)}\times {k}_{TS{aw}}$ ,${k}_{{3}^{^{\prime}}}={K}_{eq(B)}\times {k}_{TSbw}$, ${k}_{{4}^{^{\prime}}}={K}_{eq(D)}\times {k}_{TSbw}$, and are bimolecular rate coefficients of each reaction pathway involved in the OH + CH_3_OH reaction. The total rate coefficients for OH + CH_3_OH (+ H_2_O) are expressed by Eq. 15:

15 $${k}_{total-w}={k}_{Aw}^{CVT}+{k}_{Bw}^{CVT}.$$

**Figure 11:**
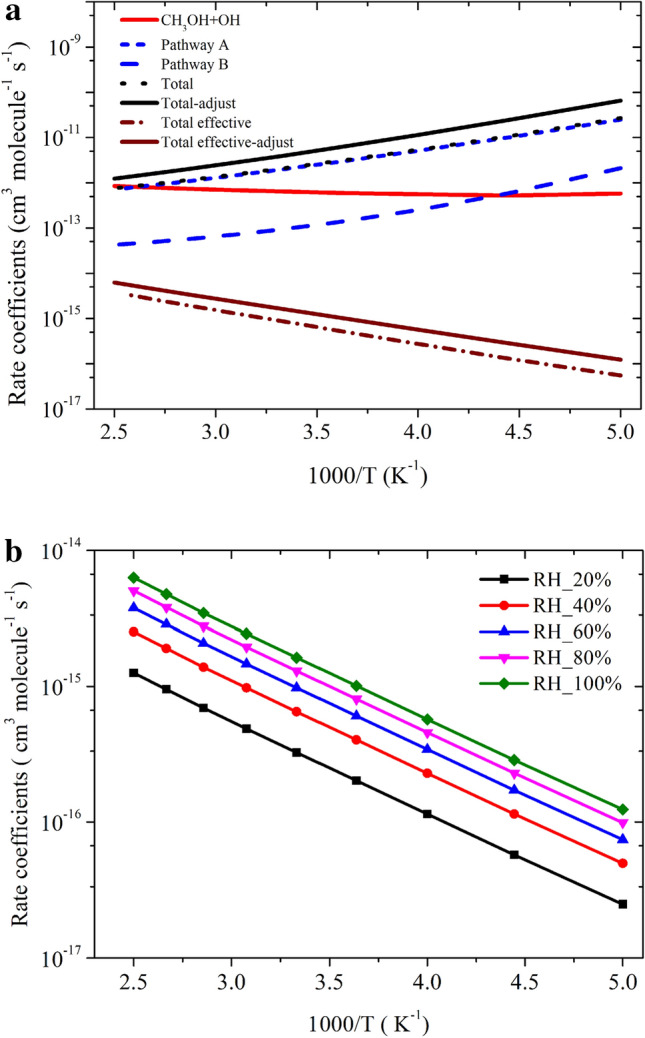
Rate coefficients for CH_3_OH + OH (+ H_2_O) (**a**) 100% relative humidity (RH), (**b**) at different RH of H_2_O.

The rate coefficients in the temperature range of 200 K to 400 K for Pathway A_*w*_, Pathway B_*w*_ and total effective rate coefficients are shown in Fig. 11. In both pathways, OH + CH_3_OH (+ H_2_O) is dominated over the H-abstraction pathway. Our results show that the rate coefficients of Pathway A_w_ are higher than Pathway B_*w*_. It can be seen that, if step 0 is ignored, the rate coefficient of CH_3_OH + H_2_O···OH reaction (1.12 × 10^–11^ cm^3^ molecule^−1^ s^−1^ at 300 K) is ~ 2 times higher than CH_3_OH + OH reaction (6.45 × 10^–13^ cm^3^ molecule^−1^ s^−1^ at 300 K). In general, the rate coefficients of water-catalyzed reaction are higher than the water-free reaction at the temperature < 300 K (see Fig. 6).

As discussed in previous studies^16–18^, the importance of step 0 cannot be neglected for the reaction under tropospheric conditions. Ignoring step 0 is equivalent to assuming that all the CH_3_OH is formed a complex with H2O, which is not true. If step 0 is taken into account, there is a negligible amount of CH_3_OH···H_2_O and H_2_O···OH and thus it is the water-free gas-phase pathway that determines the rate of the reaction. Under pseudo-first-order conditions, the relative equilibrium concentrations depend strongly on the concentration of the excess of water and the correct expression to calculate the total effective rate coefficients is given by Eq. 16:

16 $${k}_{total-w}^{eff}=\left[\left\{{K}_{eq\left(2\right)}\times {k}_{Aw}^{CVT}\right\}+\left\{{K}_{eq\left(3\right)}\times {k}_{Bw}^{CVT}\right\}\right]\times \left[{H}_{2}O\right].$$

where ${K}_{eq(2)}$ and ${K}_{eq(3)}$ are equilibrium constants of CH_3_OH + H_2_O → CH_3_OH···H_2_O, and H_2_O + HO → RC_2w_ reactions, respectively (see Table S2) and [H_2_O] is temperature-dependent water concentration based on literature value as discussed in Wu et al.^14^ The water concentration is calculated using a typical temperature-dependent water concentration, which corresponds to 100% humidity^32^. The value of [H_2_O] concentration decreases with the height of altitude. This value is in good agreement with the value of Chao et al.^11^ (8.4 × 10^–15^ cm^3^ molecule^−1^ s^−1^). Our calculated value is at least one order magnitude lower than the value reported by Wu et al.^14^ This is due to fact that they used different functional and different kinetic models. The effective rate coefficient calculated based on Eq. 16 (1.6 × 10^–15^ cm^3^ molecule^−1^ s^−1^ at 300 K) is ~ 3 order of magnitude lower than water-free OH + CH_3_OH reaction (~ 9.1 × 10^–13^ cm^3^ molecule^−1^ s^−1^ at 300 K). This is due to the water-assisted pathway depends parametrically on water concentration. Our result is also consistent with previously reported values on similar atmospheric reactions *i.e.,* OH + CH_2_NH, OH + CH_3_CHO and OH + CH_2_O^16,31,32^. Using the rate coefficients considering the water catalytic effect could decrease the atmospheric lifetime of CH_3_OH by a factor of 3 in a tropical region with high RH, which would have a non-negligible effect on the global CH_3_OH budget. It is interesting to mention that the reaction between CH_3_OH and OH in the presence of (H_2_O)_n_ n > 1 1has been studied previously and found no impact on the rate coefficients^9,12,14^. The rate coefficients were also calculated using different water concentration as shown in Fig. 11b. The effect of relative humidity from 20 to 100% on calculated rate coefficients are in ~ 1 order magnitude difference, which is consistent with the previously reported value^14^.

**Figure 12:**
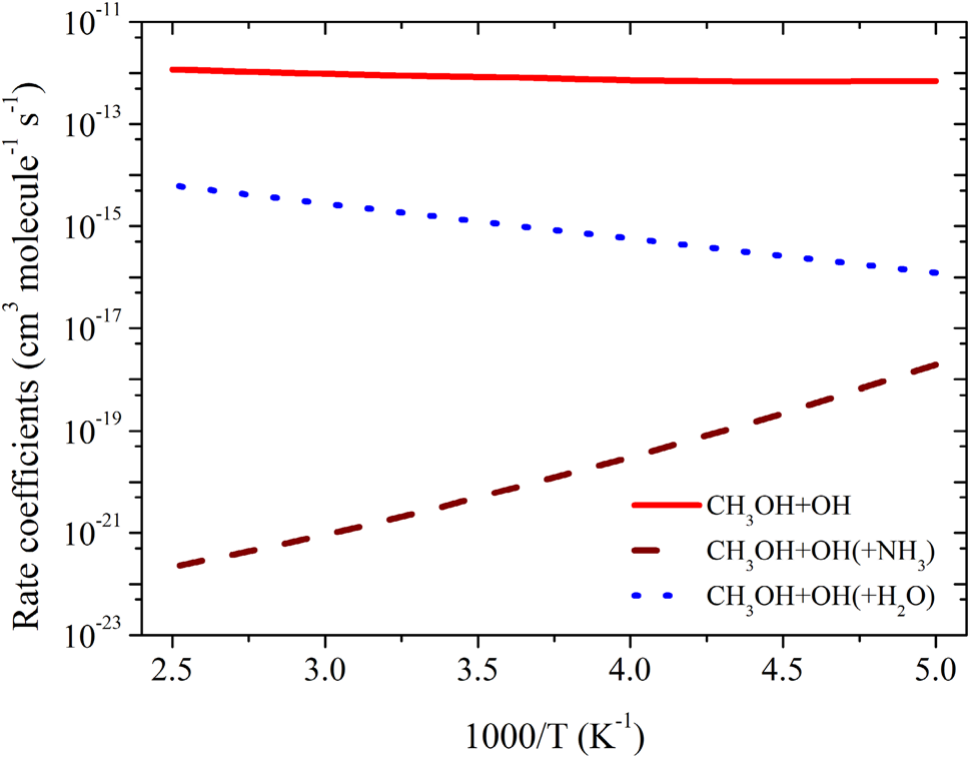
Comparison between rate coefficients for CH_3_OH + OH, CH_3_OH + OH (+ NH_3_) and CH_3_OH + OH (+ H_2_O).

The total rate coefficients and effective rate coefficients for reactions systems OH + CH_3_OH (+ NH_3_) and OH + CH_3_OH (+ H_2_O) are tabulated in Table 3 and shown in Fig. 12. The total rate coefficients for OH + CH_3_OH (+ NH_3_) (2.7 × 10^–21^ cm^3^ molecule^−1^ s^−1^ at 300 K) and OH + CH_3_OH (+ H_2_O) (1.6 × 10^–15^ cm^3^ molecule^−1^ s^−1^ at 300 K).

**Table 3:** Calculated rate coefficients for the OH + CH_3_OH, OH + CH_3_OH + H_2_O and OH + CH_3_OH + NH_3_.

Temp	${{k_{CH_3+OH}}}$	k_*effN*_	*k*_*effW*_
200	7.0 × 10^−13^	1.9 × 10^−18^	1.2 × 10^−16^
225	6.8 × 10^−13^	1.7 × 10^−19^	2.8 × 10^−16^
250	7.2 × 10^−13^	3.0 × 10^−20^	5.7 × 10^−16^
275	8.2 × 10^−13^	8.2 × 10^−21^	1.0 × 10^−15^
300	8.8 × 10^−13^	2.7 × 10^−21^	1.6 × 10^−15^
325	9.5 × 10^−13^	1.2 × 10^−21^	2.4 × 10^−15^
350	1.0 × 10^−12^	5.9 × 10^−22^	3.5 × 10^−15^
375	1.1 × 10^−12^	3.4 × 10^−22^	4.7 × 10^−15^
400	1.2 × 10^−12^	2.2 × 10^−22^	6.3 × 10^−15^
k = AT^n^ exp(− B/T)	A = 6.66 × 10^−20^ n = 2.59 B = − 488.82	A = 3.80 × 10^−52^ n = 8.90 B = − 6082.2	A = 3. × 10^−15^ n = 0.69 B = 1390.90

This value is at 8 order magnitude (in the case of ammonia) and 3 order higher (in the case of water) than OH + CH_3_OH reaction (8.8 × 10^–13^ cm^3^ molecule^−1^ s^−1^). Our calculations suggest that the catalytic effect of ammonia and water takes place if step 0 is ignored in the reaction mechanism. This result is also consistent with previous report on a similar reaction system^16–32^. If we ignore step 0, which means all the ammonia and water will be complexed with the methanol, which is not true in realistic conditions. Therefore, the correct reaction rate coefficient calculation must include the concentration of NH_3_ and H_2_O in the rate coefficient calculations. In that situation, our calculation demonstrates that the total effective rate coefficients for systems OH + CH_3_OH (+ NH_3_) (~ 6–10 order) and OH + CH_3_OH (+ H_2_O) (2–3 order) magnitude smaller than the dry situation. This result is consistent with an earlier reports on similar reaction systems^16–18^. It is clear that geometries of PRC_N_, and TSs are different in OH + CH_3_OH (+ NH_3_) reaction systems compared to its isoelectronic analogous OH + CH_3_OH(+ H_2_O) reactions, resulting in different computed enthalpies and rate coefficients. Because of this, the kinetics of OH + CH_3_OH (+ NH_3_) is quite different from those OH + CH_3_OH (+ H_2_O) reaction systems. In the case of ammonia, the rate coefficients show negative temperature-dependence and in the case of water positive temperature-dependence was observed. This result may be due to that the water concentration depends highly on temperature and ammonia concentration is nearly independent of temperature.

It is possible to extend our finding on the effect of an ammonia and water molecule to gas-phase OH + CH_3_OH, reactions. In this reaction if the concentration of the CH_3_O···X (X = NH_3_, H_2_O) the complex formed in step 0 is very low, as is the case of CH_3_O···X, the reduction of barrier height in step 2 is not expected to be large enough to increase the rate coefficients *i.e.,* a catalytic effect. As a result, the rate coefficients with ammonia (6 to10 order) and water molecules are 2 to 3 order magnitude smaller than the reaction OH + CH_3_OH reaction under tropospheric conditions. Our computations demonstrate that ammonia and water have the potential to accelerate a gas phase reaction but exhibits no enhancement under tropospheric conditions as the water-and ammonia assisted reaction is slower than the OH + CH_3_OH reaction.

### Atmospheric implications

In general, the effective rate coefficients of the ammonia and water-assisted reaction is smaller than the OH + CH_3_OH reaction system in the temperature range of 200–400 K. As a result, the effect of OH + CH_3_OH catalyzed by NH_3_/H_2_O is minor importance for the sink of CH_3_OH in gas -phase atmospheric chemistry. This result is consistent with experimental measurement and theoretically calculated reaction^11–14^. The current study of methanol in the upper troposphere has important repercussions for budget calculations. For that purpose, we have calculated the atmospheric lifetime of methanol at 225 K and (*i.e.,* at an altitude of ∼10 -11 km) taking an averaged concentration of HO radicals in the upper troposphere of OH concentration of [HO] ~ 1 × 10^6^ molecule cm^−3^. The rate coefficient at 225 K of 6.8 × 10^−13^ cm^3^ molecule^−1^ s^−1^. The lifetime for methanol of 17 days is in good agreement with the experimental value of Dillon et al. (14 days)^3^.

In the tropospheric condition, the main radical product of the reaction is CH_2_OH, which can further react with molecular ^3^O_2_ to form formaldehyde. The formation of formaldehyde may increase the budget of formaldehyde. The degradation mechanism of the loss of CH_3_OH with OH is follow as given below;

$$\begin{aligned} & {\text{CH}}_{3} {\text{OH}} +^{ \cdot } {\text{OH }} \to^{ \cdot } {\text{CH}}_{2} {\text{OH }} + {\text{H}}_{2} {\text{O}} \\ &^{ \cdot } {\text{CH}}_{2} {\text{OH}} + {\text{O}}_{2} \to {\text{ CH}}_{2} {\text{O }} + {\text{ HO}}_{2} \\ &^{ \cdot } {\text{CH}}_{2} {\text{OH}} + {\text{O}}_{2} \to^{ \cdot } {\text{OOCH}}_{2} {\text{OH}} \\ &^{ \cdot } {\text{OOCH}}_{2} {\text{OH}} \to {\text{HOOCH}}_{2} {\text{O}}^{ \cdot } \to {\text{CH}}_{2} {\text{O}} + {\text{HO}}_{2}^{ \cdot } \\ &^{ \cdot } {\text{OOCH}}_{2} {\text{OH}} \to {\text{HOO}}^{ \cdot } {\text{CHOH}} \to {\text{HHOC}} = {\text{O }} +^{ \cdot } {\text{OH}} \\ \end{aligned}$$

The atmospheric lifetime of CH_2_OH is ~ 17 ms suggest that the formation of formaldehyde is fast under atmospheric conditions. The theoretical calculation for the formation of formic acid is not known, further study is necessary to investigate possible reactions in both atmospheric and combustion reaction. Based on this study, we believe that effect of ammonia and water on CH_2_OH + O_2_ will be even slower and future study is required to understand this mechanism. In general rate coefficients for the ammonia /water-assisted reaction to form CH_2_O and CH_3_O is negligible under tropospheric condition. Therefore, the reaction in presence of water ammonia/water cannot produce CH_3_O and CH_2_O radicals under tropospheric conditions. Based on current and previous studies, we propose a rule that a single ammonia /water molecule does not catalyze the reaction of OH radicals with alcohol. If there is an exception to this rule, it remains to be found. We believe the present results provide insights into a better understanding of the gas phase catalytic effect of an ammonia and water molecule on the most important atmospheric and combustion reaction prototype molecule.

## Conclusions

In the present work, the effect of single water and ammonia molecule on the gas-phase reactions of OH + CH_3_OH has been investigated. The rate coefficients for two important reaction pathways OH + CH_3_OH···X (X = H_2_O, NH_3_) and CH_3_OH + HO···X (X = H_2_O, NH_3_) were computed calculated using CCSD(T)/6-311++G(3df,2p)//M06-2X/6-311++G(3df,2p) level with CVT/SCT approach and results were compared with previously published data.

In the presence of ammonia, the dominated reaction pathway is the H-abstraction reaction from the O–H bond. In the case of the water-assisted OH + CH_3_OH reaction system, the dominated reaction pathway is the hydrogen abstraction from the CH_3_ site. This result is true for both water-free and water-assisted reactions. Under the atmospheric condition, the kinetics of OH + CH_3_OH (+ NH_3_) is quite different from those of both OH + CH_3_OH(+ H_2_O). This catalytic difference between catalyst NH_3_ and H_2_O is possibly due to a much lower concentration of NH_3_ relative to H_2_O. Our results demonstrate that a single ammonia/water molecule has the potential to accelerate a gas phase reaction if step 0 is not included in the reaction mechanism. Ignoring step 0 is equivalent to assuming all the CH_3_OH are complexed with water, which is not true. Therefore, the correct reaction pathways should have ammonia/water concentration in the rate coefficient calculations. This result is consistent with previous studies on similar reaction systems. Despite the fact that OH + CH_3_OH reaction with the catalyst NH_3_ and H_2_O is not so efficient to shift the overall formation rate, the present study provides a comprehensive model of how basic and neutral catalysts assisted the gas-phase reactions. The atmospheric degradation mechanism suggests that the lifetime of CH_3_OH is 17 days, which can further react with O_2_ molecules to form the formaldehyde and formic acid under atmospheric and combustion conditions. The effect of ammonia and water molecules could slow the formation of formaldehyde and formic acid. Such results are interesting can be used to understand the other alcohol and similar species.

## Supplementary Information

Supplementary Information.

## Data Availability

All data generated through this study are given in Supporting Information file. Supporting Information: Tables of optimized geometries of all the species involved in the OH + CH_3_OH, OH + CH_3_OH (+ NH_3_) and CH_3_OH (+ H_2_O). Tables of Equilibrium Constants and Figure of trail rate coefficients. Energies and rate coefficients calculation using at CBS-QB3 level.
