# Supplementary material for: Effect of ammonia and water molecule on OH + CH3OH reaction under tropospheric condition
Source: Sci Rep. 2021 Jun 9;11:12185. doi: 10.1038/s41598-021-90640-6 (PMC8190139; doi:10.1038/s41598-021-90640-6)
Supplement: Supplementary file 1 — Supplementary Information. [file 41598_2021_90640_MOESM1_ESM.docx]

*****Supplementary Materials*****

**Effect of ammonia and water Molecule on OH+CH_3_OH Reaction Under Tropospheric Condition**

**Mohamad Akbar Ali*^a^, Balaganesh M.^b^, Faisal Al-Odail, ^a^ and K.C. Lin^b^**

^a^Department of Chemistry, College of Science, King Faisal University, PO box 380, Al Hufuf , 31982 Al-Ahsa, Saudi Arabia

^b^Department of Chemistry, National Taiwan University, Taiwan

Corresponding author e-mail: [aamohamad@kfu.edu.sa](mailto:akbar256@sejong.ac.kr)

*Table of Contents*

Table S1: Optimized geometries of reactants, complexes, products and transition states obtained using M06-2X/6-311++G(3df,3pd)……………………………………………….*Page 2*

***Table S2:*** *CVT rate coefficients……………………………………………………………..****Page 9***

**Table S3**: Calculated equilibrium constants (*K_eq_* in cm^3^ molecule^-1^) for the formation of two-body complexes *………………………………………………………………………………****Page 10***

**Table S4**: Calculated equilibrium constants (*K_eq_* in cm^3^ molecule^-1^) for the formation of three-body complex………………………………………………………………….. ***Page 11***

**Table S5.** Comparison of Enthalpies of reaction (∆*H_rxn_* (0 *K*) in kcal mol^-1^ of OH+CH_3_OH reaction using different level of theories………………………………………… Page 14

**Figure S1**: Trial rate constants for dissociation of CH_3_OH···HO as functions of N-O bond distance from 200 to 400 K in the interval of 25K. *…………………………………….****Page 15****.*

**Figure S2***:* IRC calculations……………………………………………………….***Page 16***

**Figure S3*:*** Relative humidity with the fucntion of temperature……………………***Page 19***

***Figure S4: Results based on CBS-QB3 level…………………………………….Page 20***

**Table S1**: Redundant internal coordinates of reactants, complexes, products and transition states obtained using M06-2X/6-311++G(3df,3pd) taken from gaussian checkpoint file.

**(a) CH_3_OH + OH==> CH_2_OH/CH_3_O + H_2_O**

**CH_3_OH**

Charge = 0 Multiplicity = 1

C,0,-0.5940673537,0.0573170656,0.0067568248

H,0,-0.2203685447,-0.9632022475,0.0280469806

H,0,-0.2330846637,0.5379750258,-0.9054315572

H,0,-1.6857927256,0.0246209537,-0.0156833147

O,0,-0.1090026726,0.6977801433,1.1682686204

H,0,-0.4210131119,1.6033301671,1.1791197064

**OH**

Charge = 0 Multiplicity = 2

O,0,0.810535131,0.88541678,0.

H,0,1.781409489,0.88541678,0.

**RC1**

Charge = 0 Multiplicity = 2

C,0,-0.659630427,0.0969321747,-0.0750165499

H,0,-0.208230856,-0.8881778791,0.0059297956

H,0,-0.3480344658,0.5509300957,-1.0160650889

H,0,-1.7445615368,-0.010571675,-0.0629129711

O,0,-0.20086383,0.8460881906,1.0385241999

H,0,-0.5680849941,1.7305522344,1.0179055311

O,0,1.5070009262,-0.7674628117,2.5736679552

H,0,0.9395205536,-0.0958096497,2.1443501382

**TS1a**

Charge = 0 Multiplicity = 2

C,0,-0.6077239871,0.0451313523,-0.0009008185

H,0,-0.3499971323,-1.0001465846,0.142445105

H,0,-0.0868709082,0.4593146838,-0.8662633582

H,0,-1.7265226124,0.0899434418,-0.2573260946

O,0,-0.3480138453,0.7294554988,1.1850808552

H,0,-0.496945432,1.6671908542,1.0472207044

O,0,-3.1777012756,0.3973587943,-0.0436543796

H,0,-3.1370790271,0.2586438693,0.9157408063

**TS2b**

Charge = 0 Multiplicity = 2

C,0,1.182348569,-0.4430600857,0.0320265455

H,0,2.1988000717,-0.0460701262,0.0171210434

H,0,1.0554651866,-1.1349841763,-0.8005577113

H,0,1.0369651333,-0.9727893026,0.9749496228

O,0,0.3274601103,0.6654475859,-0.0701995984

H,0,-0.641762795,0.3520383998,-0.36332378

O,0,-1.7075786296,-0.276922973,-0.0099816132

H,0,-1.9714570863,0.1669091381,0.8082869613

**CPa**

Charge = 0 Multiplicity = 2

O,0,-0.1990126649,0.1657414886,0.1152313728

H,0,0.2925466784,0.1201160739,0.9377358041

H,0,0.3592786765,-0.2465555776,-0.5476777839

C,0,-2.3600113727,1.9813821213,-1.689851107

H,0,-3.0051873442,2.7109378925,-2.1508636144

H,0,-2.723879068,0.9907243967,-1.4539449561

O,0,-1.3726527338,2.5025093313,-0.9284205643

H,0,-0.9164975696,1.7811136125,-0.4731658571

**CPb**

Charge = 0 Multiplicity = 2

C,0,-0.0002224707,0.000019167,0.0002686061

H,0,0.004781042,0.003270907,1.0906901528

H,0,1.0249057531,-0.0040113889,-0.3982702037

H,0,-0.4372268097,-0.9259180284,-0.39811115

O,0,-0.6576081691,1.0661584845,-0.5483867392

H,0,-0.1620388677,0.0201972917,-3.8896124896

O,0,0.0283718369,-0.2184019929,-2.9816826213

H,0,-0.3695536058,0.4714179202,-2.4369606876

**CH_2_OH**

Charge = 0 Multiplicity = 2

C,0,-1.3294976695,-0.5685219694,0.0769254257

H,0,-0.9861418954,-1.6051659422,0.0395192375

H,0,-1.0048988686,-0.0277664219,-0.8228086367

H,0,-2.4272850682,-0.5306707642,0.0484801983

O,0,-0.9010029923,0.1206141229,1.1750364713

**CH_3_O**

Charge = 0 Multiplicity = 2

C,0,-1.3294976695,-0.5685219694,0.0769254257

H,0,-0.9861418954,-1.6051659422,0.0395192375

H,0,-1.0048988686,-0.0277664219,-0.8228086367

H,0,-2.4272850682,-0.5306707642,0.0484801983

O,0,-0.9010029923,0.1206141229,1.1750364713

**H_2_O**

Charge = 0 Multiplicity = 1

O,0,-1.4346366202,0.2446432413,0.01863449

H,0,-0.4767523973,0.2816161171,0.01863449

H,0,-1.7195327818,1.1599264517,0.01863449

**Table S1**: Redundant internal coordinates of complexes, post products and transition states obtained using M06-2X/6-311++G(3df,3pd) taken from gaussian checkpoint file.

**(b) CH_3_OH + OH + (NH_3_) ==> CH_2_OH/CH_3_O + H_2_O+ NH_3_**

**RC2**

Charge = 0 Multiplicity = 2

O,0,-1.650813524,-0.0014937351,-0.0099897478

H,0,-0.6649638026,0.0011725449,-0.030436772

N,0,1.247233185,0.0002306356,-0.0708359678

H,0,1.6191198938,-0.9419872934,-0.0786547024

H,0,1.638039629,0.4709734568,0.7365261517

H,0,1.6035687088,0.4708346612,-0.8940689618

**RC3**

Charge = 0 Multiplicity = 1

O,0,0.8099537463,0.7445421749,0.3414269331

H,0,1.5379796251,1.3278501593,0.558258064

C,0,1.2975575625,-0.5769279003,0.178117818

H,0,0.4381927891,-1.1957338852,-0.0650293524

H,0,2.0212518944,-0.6394270116,-0.6364676783

H,0,1.7560167953,-0.9512542897,1.0951181702

H,0,-1.2838319403,0.4443714859,-0.031276744

H,0,-2.4812335201,0.1444157908,-1.0996767646

N,0,-2.0325573431,-0.2022275385,-0.2608616072

H,0,-2.7177333793,-0.1432299156,0.4822090913

**PRCaN**

Charge = 0 Multiplicity = 2

C,0,-1.09365812,0.14746573,-0.396615472

H,0,-0.8711895429,1.157127077,-0.0472181811

H,0,-0.4202738655,-0.0838780052,-1.2178955511

H,0,-2.1193039016,0.1059023221,-0.765138148

O,0,-0.8663574719,-0.8235294333,0.6157233793

H,0,-1.4471015809,-0.6446465316,1.3565203532

O,0,1.2919977182,1.9010342889,-1.0611769348

H,0,1.6725007421,1.1165375911,-0.5878921774

N,0,2.1141547128,-0.4283523987,0.339180994

H,0,1.2151314612,-0.7849883353,0.656459084

H,0,2.6933731534,-0.2764422057,1.1556285022

H,0,2.556577785,-1.1535458292,-0.2118868483

**PRCbN**

Charge = 0 Multiplicity = 2

O -1.4303 3.23872 -0.11789

H -2.36265 1.60859 -0.25123

C 3.00296 -0.49075 -1.02912

H 3.88306 -2.3042 -1.43096

H 4.40317 0.99764 -1.30666

H 1.45845 -0.19087 -2.375

O 2.11929 -0.56612 1.48783

H 1.24554 1.00532 1.78683

N -3.21484 -1.75077 -0.15234

H -4.64346 -2.26902 1.00998

H -1.56315 -2.32861 0.64564

H -3.44676 -2.69972 -1.79706

**TSaN**

Charge = 0 Multiplicity = 2

C 2.60369 0.03248 0.93718

H 1.26909 1.74754 0.4977

H 4.52288 0.76431 0.75493

H 2.21892 -0.57525 2.85948

O 2.09508 -1.97857 -0.69689

H 2.3936 -1.43264 -2.40093

O -0.6824 3.2826 -0.39234

H -1.98945 1.97957 -0.17066

N -3.63554 -1.27455 0.26491

H -2.05459 -2.3207 -0.04107

H -4.93823 -1.79333 -1.03617

H -4.33085 -1.74311 1.98403

**TSbN**

Charge = 0 Multiplicity = 2

O 0.45939 2.77018 0.29866

H 2.00451 1.7511 0.49163

C -3.33914 -1.09611 0.73921

H -4.49407 -2.68388 0.10203

H -4.57559 0.49 1.18661

H -2.32193 -1.6721 2.44045

O -1.68213 -0.53053 -1.24786

H -0.84963 1.36592 -0.95252

N 4.07305 -1.17321 0.20775

H 5.50705 -1.14722 -1.0579

H 2.62383 -2.15633 -0.57696

H 4.67781 -2.14022 1.74297

**CPaN**

Charge = 0 Multiplicity = 2

C 3.02504 -1.09994 0.89019

H -1.9175 3.80253 1.68744

H 2.45154 -0.36299 2.71098

H 4.60328 -2.38262 0.75313

O 2.62571 0.32948 -1.17274

H 1.30418 1.56397 -0.80358

O -1.60714 2.92528 0.13516

H -2.46824 1.28275 0.24894

N -3.04412 -2.12912 -0.12047

H -1.26001 -2.77586 0.19448

H -3.46378 -2.50436 -1.95061

H -4.2394 -3.15807 0.96206

**CPbN**

Charge = 0 Multiplicity = 2

O -0.95524 3.2666 -0.16108

H -2.02881 1.77512 -0.22877

C 3.13164 -0.62419 -0.87755

H 4.64216 -2.05772 -0.92317

H 4.01141 1.23139 -1.06349

H 1.85453 -0.99385 -2.45902

O 1.98127 -0.94193 1.42137

H -0.47275 3.40934 1.57983

N -3.47286 -1.61875 -0.19917

H -4.97473 -1.97112 0.93143

H -1.9247 -2.41513 0.60669

H -3.79519 -2.49898 -1.86636

**NH3**

Charge = 0 Multiplicity = 1

N,0,-0.1466514145,0.8304501554,0.

H,0,0.2322256868,-0.1088072227,-0.0000001076

H,0,0.2322426673,1.3000719014,0.8134168492

H,0,0.2322426673,1.3000720878,-0.8134167416

**Table S1**: Redundant internal coordinates of complexes, post products and transition states obtained using M06-2X/6-311++G(3df,3pd) taken from gaussian checkpoint file.

**(c) CH_3_OH + OH + (H_2_O) ==> CH_2_OH/CH_3_O + 2H_2_O**

**RC4**

Charge = 0 Multiplicity = 1

O,0,0.1417607921,0.4688552483,0.0359406031

H,0,-0.0619896079,-0.0323688576,0.8275424345

C,0,1.4379167837,0.1062285924,-0.4220712427

H,0,1.6316137312,0.6977571555,-1.3118152441

H,0,2.1988787983,0.3299328768,0.3258360794

H,0,1.4834766728,-0.9488694956,-0.693445709

O,0,-1.0273940357,-0.7057144004,-2.2577320293

H,0,-0.8439513187,-0.1821140832,-1.4662070002

H,0,-1.7390465981,-0.2517251007,-2.7101814989

**RC5**

Charge = 0 Multiplicity = 2

O,0,-0.0867222707,0.0638158913,-0.0625109564

H,0,-0.0855411999,0.0669486052,0.8968432747

H,0,0.8353560523,0.0855566692,-0.3264631354

O,0,-1.9094150871,1.8252379147,-1.4087937283

H,0,-1.3084124014,1.1943728933,-0.9653882769

**PRCaw**

Charge = 0 Multiplicity = 2

C,0,-0.0610653614,-0.1939728196,-0.2613829161

H,0,-0.0859377025,-0.4408099555,0.7965505412

H,0,0.9364301273,0.1683257624,-0.509838634

H,0,-0.2823552063,-1.0866702538,-0.8458938225

O,0,-1.04555534,0.8125944162,-0.4787981146

H,0,-0.9809628371,1.1382417034,-1.3775197514

O,0,0.0896971127,0.0795862822,3.2749539268

H,0,-0.3407567819,0.8791940405,2.8966020028

O,0,-1.1766452449,2.159515777,1.9231949368

H,0,-2.0725303248,2.3907977193,2.1729387697

H,0,-1.2276061249,1.8077344216,1.0180756899\

**PRCbw**

Charge = 0 Multiplicity = 2

C,0,-0.0053296844,0.0018653766,0.082423169

H,0,-0.3282245799,-0.2141491201,1.097305455

H,0,1.079252073,0.1133590323,0.0760733162

H,0,-0.2837734711,-0.8361836739,-0.560728932

O,0,-0.6489374161,1.1997723284,-0.3204386547

H,0,-0.3937791728,1.3788158565,-1.2347986806

O,0,-0.8553912071,0.9437200587,-3.2160245086

H,0,-1.7350496377,0.6731797977,-2.8654355485

H,0,-2.371940173,0.7180182639,-0.8587617982

O,0,-2.9585837281,0.3351313362,-1.5340176248

H,0,-3.8314224263,0.7039623986,-1.3928925603

**TSaw**

Charge = 0 Multiplicity = 2

C,0,-0.3769661839,0.0694750025,-0.1264683571

H,0,-1.2652633082,0.1416463665,0.493021332

H,0,0.5302481771,0.0429642728,0.4769271738

H,0,-0.4213442633,-0.9626292434,-0.6854489539

O,0,-0.3918788554,1.1246381243,-1.0474806508

H,0,0.4154653316,1.0991123194,-1.5674071086

O,0,-0.5676282607,-1.972237578,-1.6327391239

H,0,-1.2964844438,-1.5388567789,-2.1180670478

O,0,-2.3753740012,-0.0214665829,-2.7065481317

H,0,-3.2974504307,0.1641977957,-2.5239422833

H,0,-1.8595437161,0.6276617546,-2.2076286347

**TSbw**

Charge = 0 Multiplicity = 2

O,0,-0.2302392898,-0.131071585,0.3877205774

H,0,0.6602700693,-0.3720742297,0.6775081829

H,0,-0.1479735109,0.7019979589,-0.0784143144

O,0,0.2115462838,-2.8432952183,-0.2872217781

H,0,-0.2716599226,-1.9971459392,-0.3229317205

C,0,1.413877136,-2.5063007093,2.3130461369

H,0,2.1572158982,-2.141115423,3.0246962877

H,0,1.4253199978,-3.5954872247,2.3150695044

H,0,0.430929672,-2.1446204203,2.6198656388

O,0,1.784056519,-1.9874537799,1.0594975953

H,0,1.256145224,-2.5320378639,0.2563382015

**CPaw**

Charge = 0 Multiplicity = 2

C,0,-0.2396998448,-0.0416092224,-0.0431028661

H,0,-0.2181122141,0.0114622832,1.0341721434

H,0,0.5869137831,-0.4949642818,-0.57770177

H,0,-0.0369490407,-0.0947026667,-3.7844406472

O,0,-0.9146975009,0.9536001317,-0.6412958776

H,0,-0.8164517009,0.8668061571,-1.6080015486

O,0,-0.7886526155,0.0903478492,-3.2202425767

H,0,-1.1520167871,-0.7680560384,-2.9409112326

H,0,-1.4738064149,-1.65132775,-0.9675339382

O,0,-1.802241473,-2.049689757,-1.7888593811

H,0,-2.7553301171,-2.0992104063,-1.6888549124

**CPbw**

C,0,-0.089401187,0.1204667164,-0.0298062353

H,0,-0.5459561697,-0.471615898,0.7650642171

H,0,0.9588813641,0.3558895915,0.2370805621

H,0,0.0017729094,-0.4407145883,-0.9675279986

O,0,-0.6995637496,1.3243554294,-0.2211475942

H,0,2.2780355824,1.3431352502,-1.5808918634

O,0,2.8242537257,0.8162854834,-0.9767233281

H,0,3.6326352436,1.3163444883,-0.8583023719

H,0,0.1530278747,2.0815544967,-1.7429737968

O,0,0.8644055691,2.2593976657,-2.3775520078

H,0,0.5017203502,2.0872603715,-3.2474889636

**Table S2 (a)**: Calculated CVT/SCT rate coefficients (s^-1^) for OH+CH_3_OH (+NH_3_) for pathway a and pathway b.

| T (K) | *TSan* | *Tsbn* | *TSan-adjusted* | *Tsbn-adjusted* |
| --- | --- | --- | --- | --- |
| 200 | 1.08E+08 | 6.35E+07 | 1.85E+08 | 9.76E+07 |
| 225 | 2.93E+08 | 1.53E+08 | 4.82E+08 | 2.34E+08 |
| 250 | 6.91E+08 | 3.54E+08 | 1.09E+09 | 5.31E+08 |
| 275 | 1.44E+09 | 7.56E+08 | 2.20E+09 | 1.11E+09 |
| 300 | 2.69E+09 | 1.49E+09 | 3.99E+09 | 2.16E+09 |
| 325 | 4.62E+09 | 2.73E+09 | 6.67E+09 | 3.88E+09 |
| 350 | 7.41E+09 | 4.70E+09 | 1.04E+10 | 6.55E+09 |
| 375 | 1.12E+10 | 7.62E+09 | 1.55E+10 | 1.05E+10 |
| 400 | 1.62E+10 | 1.18E+10 | 2.20E+10 | 1.59E+10 |

**Table S2 (b)**: Calculated CVT/SCT rate coefficients (s^-1^) for OH+CH_3_OH (+H_2_O) for pathway a and pathway b.

| T (K) | *TSaw* | *Tsbw* | *TSaw-adjusted* | *Tsbw-adjusted* |
| --- | --- | --- | --- | --- |
| 200 | 2.45E+06 | 2.70E+05 | 6.06E+06 | 5.35E+05 |
| 225 | 6.94E+06 | 6.89E+05 | 1.59E+07 | 1.36E+06 |
| 250 | 1.66E+07 | 1.76E+06 | 3.54E+07 | 3.41E+06 |
| 275 | 3.46E+07 | 4.22E+06 | 6.95E+07 | 7.99E+06 |
| 300 | 6.46E+07 | 9.36E+06 | 1.23E+08 | 1.72E+07 |
| 325 | 1.10E+08 | 1.91E+07 | 2.01E+08 | 3.42E+07 |
| 350 | 1.75E+08 | 3.63E+07 | 3.07E+08 | 6.31E+07 |
| 375 | 2.63E+08 | 6.47E+07 | 4.45E+08 | 1.10E+08 |
| 400 | 3.77E+08 | 1.09E+08 | 6.18E+08 | 1.80E+08 |

**Table S3**: Calculated equilibrium constants (*K_eq_* in cm^3^ molecule^-1^) for the formation of two-body complexes.

###

| T(K) | *K_eq(1)_*  (CH_3_OH +OH→ RC1 | *K_eq(2)_*  (CH_3_OH +H_2_O→ RC3) | *K_eq(3)_*  (OH+ H_2_O  →RC2) | *K_eq(4)_*  (OH+ NH_3_  →RC4) | *K_eq(5)_*  (CH_3_OH+ NH_3_  →RC5) |
| --- | --- | --- | --- | --- | --- |
| 200 | 2.73×10^-19^ | 1.02×10^-20^ | 1.38×10^-19^ | 6.20×10^-19^ | 6.25×10^-19^ |
| 225 | 6.63×10^-20^ | 3.26×10^-21^ | 4.23×10^-20^ | 1.33×10^-19^ | 1.44×10^-19^ |
| 250 | 2.16×10^-20^ | 1.34×10^-21^ | 1.66×10^-20^ | 3.87×10^-20^ | 4.58×10^-20^ |
| 275 | 8.74×10^-21^ | 6.57×10^-22^ | 7.80×10^-21^ | 1.43×10^-20^ | 1.84×10^-20^ |
| 300 | 4.16×10^-21^ | 3.70×10^-22^  *6.7×10^-22^* | 4.19×10^-21^  *5.7×10^-21^* | 6.23×10^-21^ | 8.76×10^-21^ |
| 325 | 2.24×10^-21^ | 2.30×10^-22^ | 2.50×10^-21^ | 3.12×10^-21^ | 4.76×10^-21^ |
| 350 | 1.33×10^-21^ | 1.55×10^-22^ | 1.62×10^-21^ | 1.73×10^-21^ | 2.86×10^-21^ |
| 375 | 8.55×10^-21^ | 1.12×10^-22^ | 1.12×10^-21^ | 1.05×10^-21^ | 1.87×10^-21^ |
| 400 | 5.85×10^-22^ | 8.46×10^-23^ | 8.19×10^-22^ | 6.79×10^-22^ | 1.30×10^-21^ |

**Table S4**: Calculated equilibrium constants (*K_eq_* in cm^3^ molecule^-1^) for the formation of three-body complex.

| T (K) | *K_eq(A)_*  (CH_3_OH + RC2w →PRC_aw_) | *K_eq(B)_*  (CH_3_OH + RC2w →PRC_bw_ | *K_eq(C)_*  (OH + RC3_w_ → PRC_aw_) | *K_eq(D)_*  (OH + RC3_w_ → PRC_bw_) | *K_eq(E)_*  (CH_3_OH + RC4_N_ →PRC_aN_) | *K_eq(F)_*  (CH_3_OH + RC4_N_ →PRC_bN_ | *K_eq(G)_*  (OH + RC5_N_ → PRC_aN_) | *K_eq(H)_*  (OH + RC5_N_ → PRC_bN_) |
| --- | --- | --- | --- | --- | --- | --- | --- | --- |
| 200 | 8.70×10^-19^ | 6.67×10^-19^ | 9.28×10^-18^ | 7.11×10^-18^ | 5.13×10^-21^ | 1.06E-19 | 5.10×10^-21^ | 1.90×10^-21^ |
| 225 | 1.35×10^-19^ | 7.82×10^-20^ | 1.32×10^-18^ | 7.64×10^-19^ | 1.34×10^-21^ | 1.53E-20 | 1.23×10^-21^ | 3.80×10^-21^ |
| 250 | 3.07×10^-20^ | 1.42×10^-20^ | 2.77×10^-19^ | 1.28×10^-19^ | 4.70×10^-22^ | 3.28×10^-21^ | 3.97×10^-22^ | 1.04×10^-22^ |
| 275 | 9.31×10^-21^ | 3.57×10^-21^ | 7.82×10^-20^ | 3.00×10^-20^ | 2.04×10^-22^ | 9.39E-22 | 1.58×10^-22^ | 3.61×10^-23^ |
| 300 | 3.49×10^-21^ | 1.14×10^-21^ | 2.74×10^-20^ | 8.95×10^-21^ | 1.03×10^-22^ | 3.35E-22 | 7.35×10^-23^ | 1.50×10^-23^ |
| 325 | 1.54×10^-21^ | 4.37×10^-22^ | 1.14×10^-20^ | 3.24×10^-21^ | 5.91×10^-23^ | 1.41E-22 | 3.87×10^-23^ | 7.10×10^-24^ |
| 350 | 7.70×10^-22^ | 1.94×10^-22^ | 5.40×10^-21^ | 1.36×10^-21^ | 3.72×10^-23^ | 6.79×10^-23^ | 2.25×10^-23^ | 3.76×10^-24^ |
| 375 | 4.27×10^-22^ | 9.68×10^-23^ | 2.84×10^-21^ | 6.45×10^-22^ | 2.52×10^-23^ | 3.63×10^-23^ | 1.41×10^-23^ | 2.18×10^-24^ |
| 400 | 2.57×10^-22^ | 5.31×10^-23^ | 1.63×10^-21^ | 3.37×10^-22^ | 1.81×10^-23^ | 2.12×10^-23^ | 9.44×10^-24^ | 1.35×10^-24^ |

**Table S5.** Comparison of Enthalpies of reaction (∆*H_rxn_* (0 *K*) in kcal mol^-1^ of OH+CH_3_OH reaction.

| **OH + CH_3_OH →** | ***This Work*** | ***Literature****^a,b,c^* |
| --- | --- | --- |
| OH···CH_3_OH (RC1) | -4.97*,-4.57* | -4.37, -4.47,4.90, 4.82^a^ |
| HO···HCH_2_OH (TS_a_) | -0.84*,-0.35* | 0.1,0.36^a^ |
| HO···HOCH_3_ (TS_b_) | 2.50,*2.96* | 1.91,3.29^a^ |
| CH_2_OH + H_2_O | -22.39*,-22.7* | ***-23.13, 23.1,*** 23.09^b^ |
| CH_3_O + H_2_O | -14.03*,-14.5* | *-13.66,*13.73^b^*,* |
| **OH + CH_3_OH + NH_3_** → | ***This Work*** | ***Literature*** |
| OH···CH_3_OH···NH_3_ (PRC_aN_) | -10.11, -11.0 | - |
| OH···CH_3_OH···NH_3_( PRC_bN_) | -10.45,11.02 | - |
| OH··· HCH_2_OH ···NH_3_ (TS_aN_) | -6.2,-6.34 | - |
| OH···HO···HOCH_3_···NH_3_ (TS_bN_) | -4.19,-3.45 | - |
| CH_2_OH + H_2_O + NH_3_  CH_3_O + H_2_O+ NH_3_ | -22.39,-22.7  -14.03,-14.5 | - |
| **OH + CH_3_OH +H_2_O→** | ***This Work*** | ***Literature****^a^* |
| OH···CH_3_OH···H_2_O (PRC_aw_) | -10.80*,-9.85* | 10.80^c^ |
| OH··· CH_3_OH···H_2_O (PRC_bw_) | -11.52*,-10.8* | 12.03^c^ |
| OH··· HCH_2_OH···H_2_O (TS_aw_) | -6.31*,-6.10* | -4.98^c^ |
| OH···HO···HOCH_3_···H_2_O (TS_bw_) | -3.25*,-2.35* | -2.74^c^ |
| CH_2_OH + 2H_2_O | -22.39*,-22.7* | 23.09^b^ |
| CH_3_O + 2H_2_O | -14.03, *-14.5* | 13.73^b^ |

The value given in italic were calculated using CBS-QB3 level.





**Figure S1**: Computed Rate coefficients along the reaction pathways

**IRC calculation for PRC<== TSa==> CPa**





**IRC calculation for PRC<== TSb==> CPb**





**IRC calculation for PRCaN<== TSaN==> CPaN**





**IRC calculation for PRCbN<== TSbN==> CPbN**





**IRC calculation for PRCaW<== TSaW==> CPaW**





**IRC calculation for PRCbW<== TSbW==> CPbW**





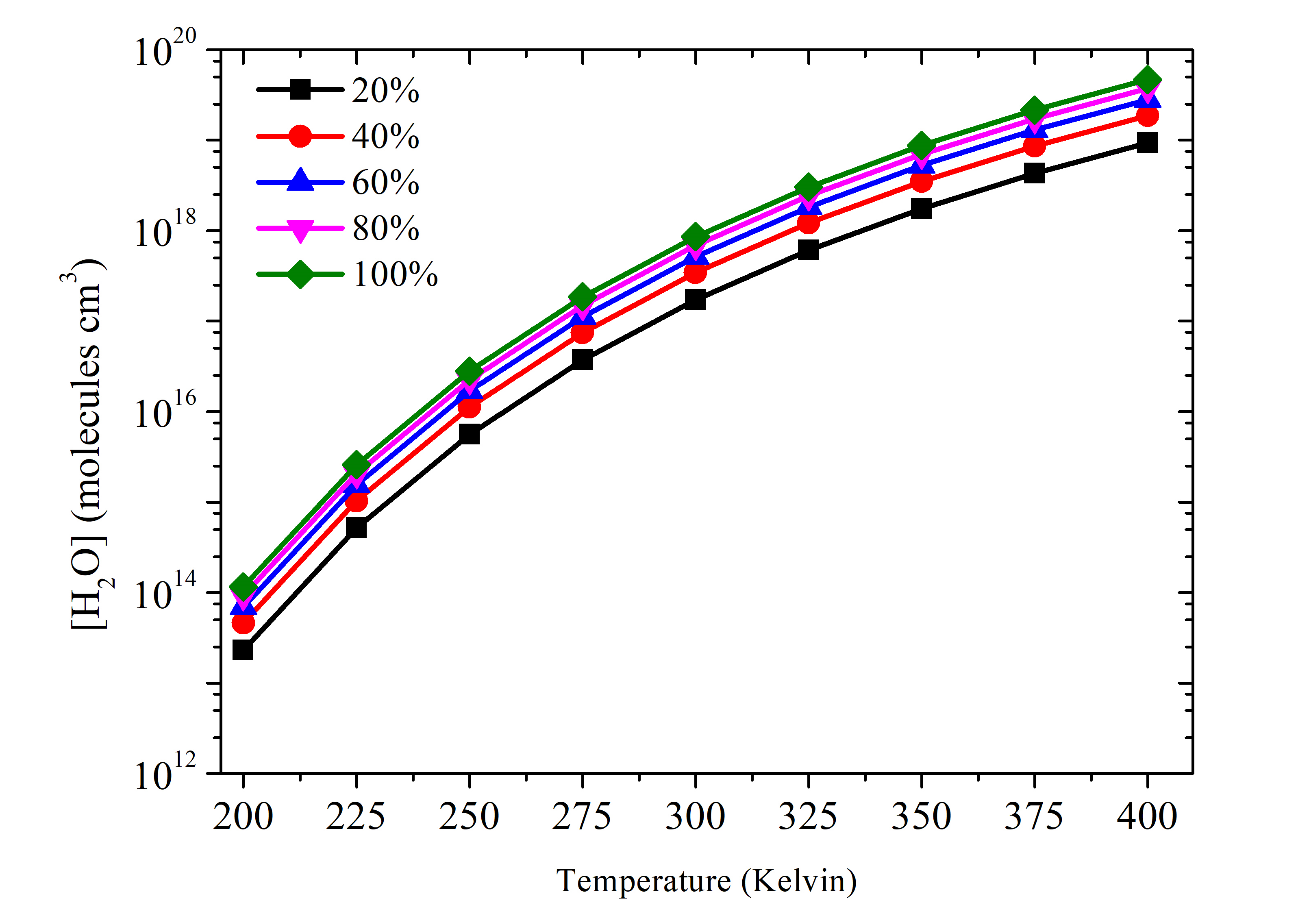


**Figure S3**: Water Concentration as a function of Temperature and relative humidity. The calculation was obtained from Reference 14.

**Rate Coefficients calculation based on CBS-QB3 level.**


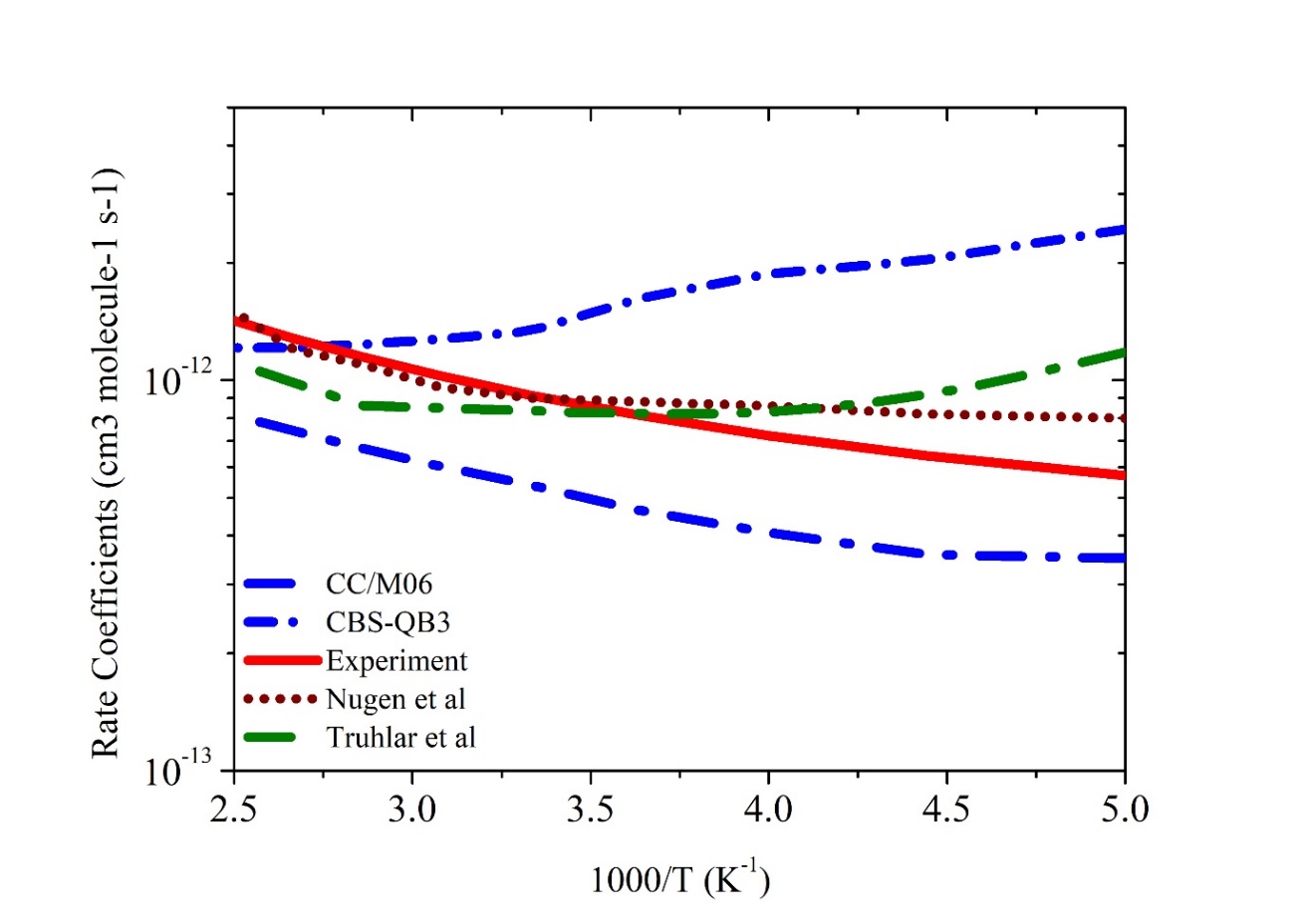


**Figure S4**. Rate coefficients for OH+CH_3_OH reaction.


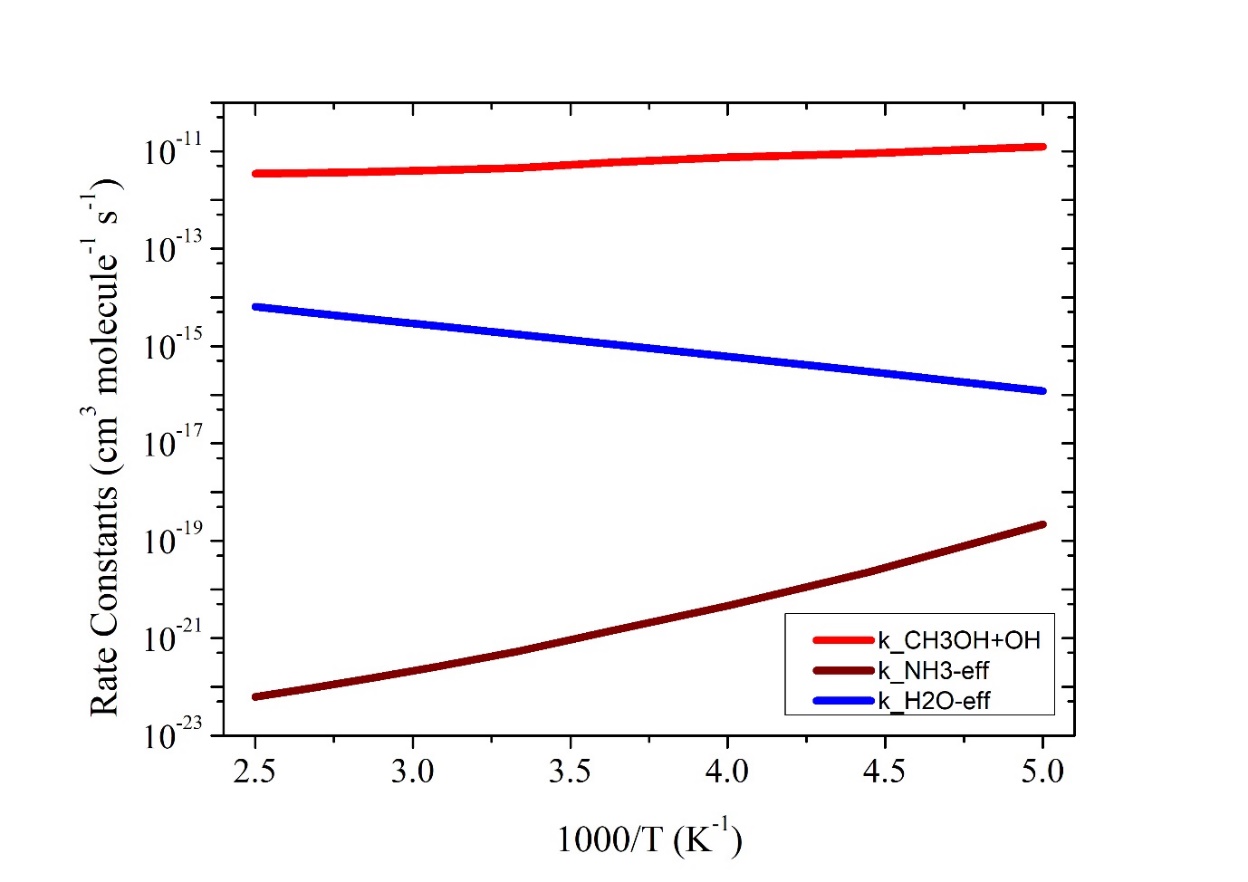


**Figure S5**. Comparison between rate coefficients for CH_3_OH+ OH, CH3OH+OH (+NH_3_) and CH3OH +OH (+H2O) calculated using CBS-QB3 level.
